# Bacteriophage-mediated cell lysis externalizes a metabolically valuable nutrient to broadly modulate bacterial communities

**DOI:** 10.1093/ismejo/wrag160

**Published:** 2026-06-23

**Authors:** David da Silva Barreira, Hannah M Poole, Rogério A Bataglioli, Bryan B Hsu

**Affiliations:** Department of Biological Sciences, Virginia Tech, Blacksburg, VA 24061, United States; Fralin Life Sciences Institute, Virginia Tech, Blacksburg, VA 24061, United States; Center for Emerging Zoonotic, Arthropod-borne Pathogens, Virginia Tech, Blacksburg, VA 24061, United States; Department of Biological Sciences, Virginia Tech, Blacksburg, VA 24061, United States; Fralin Life Sciences Institute, Virginia Tech, Blacksburg, VA 24061, United States; Center for Emerging Zoonotic, Arthropod-borne Pathogens, Virginia Tech, Blacksburg, VA 24061, United States; Department of Biological Sciences, Virginia Tech, Blacksburg, VA 24061, United States; Fralin Life Sciences Institute, Virginia Tech, Blacksburg, VA 24061, United States; Center for Emerging Zoonotic, Arthropod-borne Pathogens, Virginia Tech, Blacksburg, VA 24061, United States; Department of Biological Sciences, Virginia Tech, Blacksburg, VA 24061, United States; Fralin Life Sciences Institute, Virginia Tech, Blacksburg, VA 24061, United States; Center for Emerging Zoonotic, Arthropod-borne Pathogens, Virginia Tech, Blacksburg, VA 24061, United States

**Keywords:** phage-mediated lysis, cobamides, bacterial communities

## Abstract

Cobamides, including vitamin B12, are essential cofactors exchanged between organisms across diverse ecosystems including oceans, soils, and the mammalian gut. Although most organisms depend on cobamides, only a minority of prokaryotes perform their metabolically costly biosynthesis. This has led to vitamin B12 uptake from the environment as a common acquisition strategy, but does not explain why B12 producers would allow B12 to become extracellularly available. Our work reconciles this inconsistency, showing that bacteriophages (phages) can facilitate the release of intracellular B12 at physiologically relevant concentrations. Phages are viruses that infect and often lyse their targeted bacteria, which may externalize intracellular material along with progeny phages. In this work, we aimed to determine whether phage-mediated lysis promotes the externalization of vitamin B12, thereby supporting the growth of B12-dependent bacteria. To test this, we first used genetically well-defined B12-Producer and B12-User bacteria, and found that phage-mediated lysis of the producers releases sufficient B12 to support the growth of the users in co-culture. Next, we showed that phage-mediated lysis of the B12-producer can similarly support the growth of various commensal gut bacteria that are also B12-dependent, in co-culture. B12 released by phage-mediated cell lysis induced significant compositional changes among the non-targeted bacteria including an increased diversity that was muted by supplementing B12 in the medium. Collectively, these findings suggest that phage-mediated bacterial lysis is a significant contributor to nutrient externalization in microbial communities, leading to broad compositional changes beyond their host range.

## Introduction

Bacteriophages (phages) are among the most abundant biological entities on Earth, with an estimated 10^30^ particles infecting ~10^23^ cells every second [1]. They are active across diverse environments with up to 10^11^ viral-like particles (VLP) per liter of seawater [2] and up to 10^10^ VLP per gram of human feces [3, 4]. Studies have shown that the introduction of phages in microbial ecosystems can lead to changes in bacterial composition; this has been observed with *in vitro* culture models [5, 6] and *in vivo* mouse models [7, 8]. Such effects could also be observed when defined phage formulations of single to multiple phages were administered to animal models of gut microbiomes [9–11]. By contrast, other studies have shown that the effect of phages is less significant *in vitro* [12] and *in vivo* [13–15]. There is a notable lack of consensus regarding the breadth of effect that phages can have in gut-associated microbiomes. We hypothesize that there are additional underappreciated factors that can affect gut microbiomes that cannot be purely described by changes in bacterial concentration.

Killing of a bacterial host is not the sole consequence of infection by a lytic phage. In the world’s oceans, phage predation is estimated to lyse 20%–40% of surface bacteria daily [16], with the released intracellular material responsible for up to an estimated quarter of the dissolved organic carbon present in oceans, substantially modulating the flux of organic matter as well as biogeochemical cycles [17, 18]. It is clear that dietary alterations can substantially modulate the structure of the microbial community in the mammalian gut [19] and so we posit that the intracellular materials released by phage-mediated lysis could similarly alter the gut microbiota. Evidence of the potential significance of microbial metabolites externalized by phage can be gleaned from the importance of cross-feeding, where metabolites produced by one species are taken up for metabolism by another species [20, 21], which occurs to varying extents in all ecosystems (e.g., oceans, soils, and the gut microbiome) [21, 22]. Bacteria that develop a reliance for metabolite acquisition from the environment can gain a competitive advantage within complex communities because they can shed genomically encoded and energetically costly metabolic pathways associated with the metabolite’s biosynthesis (“genomic streamlining”) [23]. There can be additional benefits such as enhanced cooperativity with other species that have complementary abilities for metabolite production or acquisition from the environment, leading to a greater community stability [24, 25]. The benefits of cross-feeding may even extend beyond the microbial community, as loss of cross-feeding interactions was linked to 10 out of 11 diseases, including Crohn’s disease, in a computational analysis using metagenome-scale metabolic models [26]. Although there is evidence that many types of metabolites are cross-fed between microbes, the mechanisms of their release to the extracellular environment have been elucidated for only a few of these compounds [27]. Based on the prevalence and abundance of phages within naturally occurring microbial communities and the potential impact that phages can have in the structure of such communities, we speculate that phage-mediated bacterial lysis is a major facilitator for the externalization of intracellular materials that can be acquired by other bacteria [28, 29].

Vitamin B12 is a central molecule in trophic exchanges within marine and soil ecosystems as well as the gut microbiome [30–32]. Out of 11 000 publicly available bacterial genomes, 86% of bacterial species are found to encode B12-dependent processes, despite only a third encoding complete pathways for its de novo biosynthesis [33–35], indicating that a large fraction of bacteria must acquire B12 from the environment. Because B12 is produced only by bacteria and archaea, mammals must similarly obtain it from the environment or their diet [36, 37]. Cobamides, the family of compounds to which B12 belongs, are characterized by a structurally conserved corrin ring that chelates a cobalt ion with variable upper and lower ligands [38]. Although “vitamin B12” generally refers to cyanocobalamin [39, 40], for simplicity this term will refer to all bioactive cobamides including the cyanocobalamin we use for medium supplementation.

B12 is an important cofactor in several metabolic pathways including nucleotide synthesis, amino acid metabolism, and overall carbon/nitrogen metabolism [41]. This is especially the case for gut bacteria, which use B12 as a cofactor for the B12-dependent methionine synthase (MetH) that converts homocysteine to methionine [42]. The *de novo* synthesis of B12 can require 30 genes [43] and thus general strategies for B12 acquisition can be through the recycling of precursors or its acquisition from the environment [34]. In humans, *Bacteroides* represent a major (20%–80%) bacterial constituent of the gut microbiome that use B12 [35] and encode multiple B12 transporters to expand the breadth of B12 analogs they can acquire from the environment, maximizing their competitiveness [44]. This prevalence for B12 acquisition from the environment suggests that appreciable quantities are available extracellularly for the capture and uptake by other bacteria. Within microbial communities, only a small subset of bacteria encode complete B12 biosynthesis pathways [33]. In soil, these B12-Producers act as keystone species by providing sufficient extracellular B12 to sustain large communities of B12-dependent users [45]. However, among bacteria and archaea that exclusively produce B12 [35], there is little evidence for active B12 export [21, 36]. This incongruence between known mechanisms of export *versus* import is similarly a conundrum for marine and soil bacteria [46, 47]. Beyond B12, this leads to a greater question of how and why bioenergetically valuable compounds become extracellularly available [27].

In naturally occurring ecosystems, many microbially produced nutrients are shared between those that produce them and those that must acquire them for growth, but the mechanisms by which nutrients are released from the producers is known for only a few compounds. In the case of B12 where there is no known exporter, we hypothesize that phages are critical for mediating its sharing. Herein, we show that phage-mediated cell lysis of a B12-producing bacteria leads to its externalization and subsequent utilization by co-cultured bacteria that must acquire B12 for growth. In a bacterial consortium, this phage-mediated bacterial lysis leads to compositional alterations that are dependent on both the amounts of B12 released from prototrophs and its concentrations in the environment, but not the killing of the phage-targeted species. Collectively, our work describes a mechanism by which phages can broadly impact bacterial species they do not directly target and explains a mechanism by which significant quantities of a metabolically valuable metabolite can become available extracellularly for acquisition by other organisms.

## Materials and methods

### Bacterial strains and growth conditions

The bacterial strains used in this study are detailed in Table S1.

For co-culture experiments, *Escherichia coli* K-12 BW25113 and *Salmonella enterica* Typhimurium 14 028 s (Se) were pre-cultivated in lysogeny broth (LB) for 24 h from a single colony and then washed three times with NaCl solution before being grown in minimal M9 medium (Na_2_HPO_4_ [42 mM], KH_2_PO_4_ [22 mM], NH_4_Cl [20 mM], NaCl [8.5 mM], D-glucose [0.5%], CaCl2 [0.5 mM], and MgSO4 [1 mM]). Medium was supplemented with or without vitamin B12 (cyanocobalamin) or L-methionine.

Unless otherwise specified, *Bacteroides fragilis* JCM 11017 (Bf), *Bacteroides intestinalis* JCM 13265T (Bi), *Bacteroides ovatus* JCM 5824T (Bo), *Clostridium sporogenes* ATCC 17866 (Cs), *Escherichia coli* Nissle 1917 (Ec) were cultured under strict anoxic conditions in brain heart infusion-supplemented (BHIS) (ATCC medium 1293). Bacteria were maintained in a 10% (vol/vol) CO2, 2% (vol/vol) H2/N2 atmosphere at 37°C in a Coy Lab’s vinyl anoxic chamber.

For community co-culture experiments with Bo, Bi, Bf, Cs, Ec, and Se, all bacteria were pre-cultured in liquid BHIS for 24 h under strict anoxic conditions from a single colony. After three washes with NaCl solution, cells were grown in a Defined Deficient of B12 (DDB) medium supplemented or not with vitamin B12 (cyanocobalamin) or L-methionine. Each bacterial strain (Bo, Bi, Bf, Cs, Ec), and one of the Se-Producer strain: Δ*res,* Δ*res* Δ*cbiB* and Δ*res* Δ*cysG* was inoculated at an initial optical density of 0.01 at 600 nm (OD600) with active or heat-inactivated (100°C, 15 min) phage P22H5 at a multiplicity of infection (MOI) of 0.0001. Samples were collected after 24 h by centrifugation (5 min at 8000 g) and prepared for 16S rRNA gene sequencing (see below). When T4 was used, a similar experimental setup was used where the bacterial community (Bo, Bi, Bf, Cs, Ec, and Se Δ*res*) was cultured under identical conditions with either: active or heat-inactivated phage T4 at an MOI of 0.01, or, ampicillin at low (5 μg/ml) or high (50 μg/ml) concentrations before collection and preparation for 16S rRNA gene sequencing.

Composition of the DDB medium was adapted from prior studies [48, 49]. The medium contained salts (KH_2_PO_4_ [6.625 mM], NaCl [15.4 mM], CaCl_2_·2H_2_O [0.182 mM], MgCl_2_·6H_2_O [0.1 mM], MnCl_2_·4H_2_O [50.5 μM], CoCl_2_·6H_2_O [4.2 μM], NH_4_Cl [9.35 mM], Na_2_SO_4_ [1.76 mM], FeSO_4_·7H_2_O [7.2 mM], and (NH_4_)_2_SO_4_ [300 μM]); various carbon and nitrogen sources (D-Glucose [27.7 mM], cysteine hydrochloride ·H20 [2.85 mM], and L-tryptophan [2.5 mM]), amino acid mix minus (methionine (D9517-03C) [0.5%], uracil [90 μM], adenine monohydrochloride [58 μM], guanine hydrochloride [54 μM], xanthine [65 μM], and NaHCO_3_ [0.2%]); vitamins and hemes (hemin [2 μM], vitamin K1 [1 μL/L], thiamin hydrochloride (B1) [3 μM], riboflavin (B2) [2.7 μM], nicotinamide (B3) [8.2 μM], calcium-D-pantothenate (B5) [4.2 μM], pyridoxine hydrochloride (B6) [5 μM], biotin (B7) [4.1 μM], folic acid (B9) [2.3 μM], and p-aminobenzoic acid (B10) [7.3 μM]).

The potential of hydrogen (pH) of the DDB medium was adjusted to 7.1 with a 1 M NaOH solution.

### Bacteriophage amplification and preparation of high-titer stocks

High-titer phage stocks of P22H5 were obtained by concentration *via* polyethylene glycol (PEG) precipitation. In short, host strains *S.* Typhimurium were inoculated at 37°C in LB medium (supplemented with 10 mM CaCl_2_) at an initial OD600 of 0.1. Phages P22H5 were added at a MOI of 0.1 when the bacteria reached an OD600 of 0.5. Phage amplification was allowed for 3 h before lysates were harvested by centrifugation and filtration through a 0.45 μm filter. NaCl was dissolved to a final concentration of 0.5 M to promote dissociation of phage particles from bacterial debris. After 1 h at 4°C, PEG6000 was added to a final concentration of 10% and phages were allowed to precipitate overnight at 4°C. Phage particles were harvested in phage buffer after centrifugation at 10 000 g for 15 min. Finally, PEG and bacterial debris were removed by extraction with an equal volume of chloroform, and the aqueous phase containing high-phage titer were filtered using a 0.45 μm filter.

The phage P1 and P22 HT105/1 int-201 were propagated in their respective host either in liquid LB medium (supplemented with 10 mM CaCl_2_) or using the double agar overlay method. In the latter, phages were harvested from soft-agar (0.5%) after resuspension in phage buffer and filtration (0.45 μm).

All phages were stored in phage buffer at 4°C.

### Construction of mutant strains

*Salmonella enterica* Typhimurium single mutant Δ*metH* was generated using Lambda–Red mutagenesis as described in detail elsewhere [50]. In short, a kanamycin resistance cassette flanked with 50 base pair (bp) homologous overhangs was amplified by PCR using gene-specific primers as detailed in Table S1. The PCR products were then purified by gel extraction and transformed by electroporation into competent *S.* Typhimurium cells expressing the Lambda Red recombinase (carried by the plasmid pKD46). Mutants were selected on solid LB agar plates supplemented with kanamycin (50 μg/ml).

The triple mutants of *S*. Typhimurium 14 028 s were obtained by P22 HT105/1 int-201 transduction of single mutants available in the McClelland collection [51]. The antibiotic resistance cassettes in recipient strains were removed through recombination between FLP Recognition Target sites using the Flippase (FLP) recombinase (carried in the plasmid pCP20), as previously described [52]. This allowed for selection of recombinant colonies carrying donor resistance cassettes.

Similarly, the double mutant *E. coli* Δ*metE*::KanR Δ*metH* was obtained by P1 transduction using single mutants available in the Keio collection [53]. All mutants were selected on LB agar plates supplemented with kanamycin (50 μg/ml) or chloramphenicol (25 μg/ml) depending on the resistance cassette carried by donor strains.

### Bacteriophage titration and cell counting

Phage titers were determined using the soft-agar overlay technique. First, molten top agar (LB with 0.5% agar and 10 mM CaCl_2_ at 45°C) was mixed with 100 μL of an overnight culture of the host bacteria (*S*. Typhimurium and poured onto plates of LB plates (1.5% agar). After solidifying, 5 μL of 10-fold serial dilutions done in phage buffer were spotted onto the top agar plates and incubated at 37°C overnight. Finally, phage titers were calculated and expressed as plaque-forming units per milliliter (PFU/ml).

Bacterial concentrations were determined by the spot assay method. In brief, bacterial suspensions were serially diluted 10-fold in a NaCl solution and spotted on selective LB plates, either with 50 μg/ml kanamycin or 25 μg/ml chloramphenicol. Bacterial counts were expressed as colony-forming units per milliliter (CFU/ml).

### Co-culture Duet system

A co-culture system (Cerillo) was used to monitor nutrient exchanges over time between Producer and User strains. A co-culture Duet connects two wells *via* a semi-permeable membrane (0.45 μm filter), allowing nutrient diffusion while maintaining bacteria in separate compartments. One-half of the duet was inoculated with Se-Producers at an OD600 of 0.5, with or without active phage P22H5 (MOI 1). The other half was inoculated with Se-Users (Se Δ*rfbP* Δ*metE* Δ*cbiB*, Se Δ*rfbP* Δ*metE* Δ*metH* and Se Δ*rfbP*) at an OD600 of 0.05. The simultaneous growth of each different strains in the duet was measured over time by optical density at 600 nm using a plate reader.

### Mechanical and phage lysate preparation

Mechanical lysis was performed by bead beating for 10 min at 4000 rpm with 0.1 mm silica beads (ref. D1131-01). Briefly, bacteria were washed three times with a NaCl solution (successive cycle of centrifugation, 5 min at 5000 g) and then resuspended to an equivalent OD600 of 4, though measured from a diluted suspension, before being mechanically lysed by agitation with silica beads. Cell debris was removed by centrifugation at 5000 g for 5 min, followed by filtration through a 0.2 μm filter.

Phage lysates were obtained by infecting *S.* Typhimurium strains with the phage P22H5. The different strains of *S*. Typhimurium were first inoculated in DDB medium at an initial OD600 of 0.5 followed by infection with a MOI of 1. After 4 h of infection at 37°C, cell lysates were collected after removal of debris by centrifugation (5000 g for 5 min) and filtration through a 0.2 μm filter.

Mechanical and phage lysates were stored at −80°C.

### B12 and methionine bioassays

Cobalamin (B12) concentration in samples were quantified using a bioassay relying on the growth of the *Lactobacillus delbrueckii* subsp. *lactis* ATCC 7830 (formerly *Lactobacillus leichmannii*) as a test organism. The bioassay was performed with the vitamin B12 assay medium (B3801) following the manufacturer’s instructions with some modifications. *L. delbrueckii* can substitute cobalamin with nucleotides and deoxyribonucleotides (known to be alkali-resistant factors) [54, 55]. To account for growth due to alkali-resistant factors and avoid overestimation of B12 concentration, samples were analyzed before and after alkaline hydrolysis. The amounts of B12 were calculated by subtracting growth with the alkali-resistant factor from the total growth measured by optical density at 620 nm. Alkaline hydrolysis of vitamin B12 was performed by the addition of NaOH to a final concentration of 0.2 M and incubation at 100°C for 30 min. Samples were neutralized with 0.2 M of HCl before bio-quantification.

Similarly, methionine concentrations were analyzed by measuring the growth of an *E. coli* methionine auxotrophic strain (*ΔmetE::Kan ΔmetH*) as a test organism. The assay was performed in minimal M9 media (see composition above) at 37°C under vigorous agitation for 20 h. Cells were incubated with tested samples and calibration standards. Finally, growth was measured by optical density at 600 nm.

### 16S rRNA gene sequencing and diversity analysis of bacterial communities

Bacterial community composition was determined by 16S rRNA gene V3–V4 targeted sequencing using the ZymoBIOMICS Targeted Sequencing Service (Zymo Research). Samples containing approximately 10^9^ cells were prepared in DNA/RNA Shield and processed for DNA extraction with the ZymoBIOMICS-96 MagBead DNA Kit (Zymo Research, Irvine, CA) on an automated platform. Library preparation used the Quick-16S next-generation sequencing Library Prep Kit (Zymo Research, Irvine, CA) to amplify the V3–V4 region of the 16S rRNA gene. The sequencing library was prepared using a process in which PCR amplifications were performed in real-time PCR machines to control cycles and therefore limit PCR chimera formation. The final PCR products were quantified with quantitative PCR fluorescence readings and pooled together based on equal molarity. The final pooled library was cleaned with the Select-a-Size DNA Clean & Concentrator (Zymo Research, Irvine, CA), then quantified with TapeStation (Agilent Technologies, Santa Clara, CA) and Qubit (Thermo Fisher Scientific, Waltham, WA). Controls included the ZymoBIOMICS Microbial Community Standard (Zymo Research, Irvine, CA) used as a positive control for each DNA extraction. The ZymoBIOMICS Microbial Community DNA Standard (Zymo Research, Irvine, CA) was used as a positive control for each targeted library preparation. Negative controls (i.e. blank extraction control, blank library preparation control) were included to assess the level of bioburden carried by the wet-lab process. Sequencing was performed on a NextSeq2000 System (Illumina) with a P1 reagent kit (600 cycles). The sequencing was performed with 30% PhiX spike-in. Bioinformatics analysis involved the DADA2 pipeline for sequence variant inference and error/chimera removal [56], taxonomy assignment via Uclust (Qiime v.1.9.1) against the Zymo Research Database, a 16S rRNA gene database, i.e. internally designed and curated, as reference. Composition visualization, alpha-diversity, and beta-diversity analyses were performed with Qiime v.1.9.1 [57]. If applicable, taxonomy that have significant abundance among different groups were identified by linear discriminant analysis effect size (LEfSe) [58] using default settings. To determine absolute bacterial abundance in 16S rRNA gene sequencing, cell density was used to quantify the total microbial load in the sample as previously described [59]. The absolute abundance for each bacterial species was calculated by multiplying the relative abundance by the total microbial quantity. Shannon Index indices were determined based on the abundance of bacterial species across experimental conditions. Analyses were performed using base R and the “vegan” package available in R.

### Statistical analysis

All data points represent independent experiments; results were calculated from a minimum of three biological replicates. Statistical analyses were performed using GraphPad Prism (GraphPad Software).

Error bars represent the standard deviation (SD) with ^*^ indicating that *P* values are lower than .05. *P* values lower than .05 were considered significant.

## Results

### Se produces B12 and can be engineered to require it for growth

**Figure 1:**
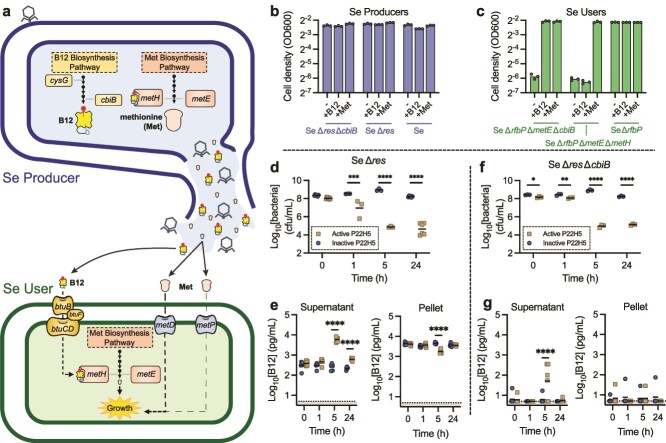
Bacteria release and acquire extracellular B12. (a) Schematic representation of the co-culture system used to study phage-mediated externalization of B12. When the Se-Producer is lysed by P22H5 phage, the intracellular content including B12 is released to the extracellular environment. The Se-User measures the utilization of the released B12 and other nutrients. (b) Strains of Se-Producers and (c) Se-Users were cultured under oxic conditions in M9 medium alone or supplemented with cyanocobalamin (B12) or methionine (Met) at 37°C for 24 h. Bacterial density was then measured by optical density at 600 nm (OD600). (d) Cultures of Se Δ*res* and (f) Se Δ*res* Δ*cbiB* were inoculated under strict anoxic conditions with active or heat-inactivated P22H5 phage at a starting OD of 0.5 and MOI of 1. (e) The concentration of B12 present in the supernatant or cell pellet of Se Δ*res* and (g) Se Δ*res* Δ*cbiB* was measured at each time point. Statistical significance was determined by two-way ANOVA or a mixed-effects model with a Bonferroni multiple comparisons test (^*^, *P <* .05; ^**^, *P <* .005; ^***^, *P <* .0005; ^****^, *P <* .0001).

To test our hypothesis that phage-mediated bacterial lysis can contribute to the bioavailability of vitamin B12 in the environment, we used *S. enterica* Typhimurium (“Se-Producers”), which synthesizes predominantly pseudo-B12 under strictly anoxic conditions, a form of corrinoid characterized by adenine as the lower α-ligand [60, 61]. We inserted a kanamycin resistance cassette into the *res* gene to enable the selective quantification of Se-producer strains (Table 1). We also generated Se mutants that are deficient in B12 biosynthesis and must acquire it from the environment for growth (“Se-Users”) (Fig.  1a; Table 1). When distinguishing the metabolic effect of B12, which is a cofactor required for the final step of methionine biosynthesis in Se, we needed to also consider potential confounding effects from bioavailable methionine. Therefore, to distinguish the effect of B12 from other metabolites released by a wildtype Se-Producer (Se Δ*res*), we used a *cbiB* mutant, i.e. unable to convert adenosylcobyric acid (AdoCby) to adenosylcobinamide phosphate, which is an essential step for the completion of B12 biosynthesis in Se [62]. This mutant Se-Producer, Se Δ*res* Δ*cbiB*, is able to produce and release Met (methionine) and the biosynthetic precursors of B12 up to AdoCby, but not the later metabolites that lead to the complete form of B12 [40, 62] (Fig.  1a). The *res* gene encodes a well-characterized enzyme associated with Restriction-Modification that has little to no impact on bacterial growth or phage P22 sensitivity.

**Table 1:** Characteristics of producer and user strains.

Strains	Abbreviation	Genotype	Growth requirements	Resistances	Phenotype
*Salmonella enterica* Typhimurium 14 028 s					
“Se-Producers”	SeΔ*res*	Δ*res*::Cm	-	Cm^R^	Biosynthesizes B12 and methionine
	SeΔ(*res,cbiB*)	Δ*cbiB,* Δ*res*::Cm	-	Cm^R^	Incomplete B12 biosynthesis (unable to convert adenosylcobyric acid to adenosycobinamide)
	SeΔ(*res,cysG*)	Δ*cysG,* Δ*res*::Cm	-	Cm^R^	Incomplete B12 biosynthesis (unable to convert uroporphyrinogen III to precorrin-2)
					
“Se-Users”	SeΔ(*rfbP,metE,cbiB*)	Δ*metE,* Δ*cbiB,* Δ*rfbP*::Kan	B12 or Met	Kan^R^	Must acquire methionine or B12 for growth
	SeΔ(*rfbP,metE,metH*)	Δ*metE,* Δ*metH,* Δ*rfbP*::Kan	Met	Kan^R^	Must acquire methionine for growth; cannot utilize B12
	SeΔ*rfbP*	Δ*rfbP*::Kan	-	Kan^R^	Does not need to acquire B12 or methionine for growth
*Escherichia coli* K-12 BW25113					
“Ec-Users”	EcΔ*metE*	Δ*metE*::Kan	B12 or Met	Kan^R^	Must acquire methionine or B12 for growth
	EcΔ(*metE,metH*)	Δ*metE*::kan ΔmetH	Met	Kan^R^	Must acquire methionine for growth; cannot utilize B12
	EcΔ*ars*	Δ*arsB*::kan	-	Kan^R^	Does not need to acquire B12 or methionine for growth

We generated a Se-User by starting with a *cbiB* mutant unable to make B12, and deleted the *metE* gene, which encodes a B12-independent methionine synthase. Bacterial growth and methionine (Met) biosynthesis is now solely dependent on the B12-dependent methionine synthase, encoded by the *metH* gene [63]. Thus, acquisition of Met by this *cbiB,metE* mutant must occur by either acquisition of extracellular Met or extracellular B12 as a cofactor for the B12-dependent methionine synthase (Se Δ*rfbP* Δ*metE* Δ*cbiB*) (Table 1). Presently, Met import mechanisms in Se are not completely described [64] and so we are unable to generate a Se-User control strain that uses B12 but not Met. However, we generated a *metE* and *metH* mutant that must acquire Met from the environment, and does not utilize B12 (Se Δ*rfbP* Δ*metE* Δ*metH*) (Fig.  1a).

In anticipation of co-culture experiments where we would introduce the obligately lytic Se phage, P22H5, we deleted the *rfbP* gene in Se-Users. This gene encodes for a galactosyl transferase, i.e. involved in the initial stages of *O*-polysaccharide synthesis in lipopolysaccharide and is required for P22 phage infection [65]. We then validated the growth requirements of our Se strains by culture in M9 minimal medium supplemented with either 100 pg/ml of B12 or 100 μM of Met. These concentrations in M9 medium were chosen based on titration curves (Fig. S1). We found that Se-Producers grew robustly under oxic conditions, regardless of medium supplementation (Fig.  1b), and Se-Users had the expected growth deficiencies; B12 and Met supplementation rescued Se Δ*rfbP* Δ*metE* Δ*cbiB*) growth and only Met rescued (Se Δ*rfbP* Δ*metE* Δ*metH*) growth (Fig.  1c). A summary of strains genotype and relevant phenotypes is presented in Table 1.

### Phage-mediated lysis of bacteria releases B12

To evaluate whether significant quantities of B12 are released from Se-Producers upon lysis by phage, we mixed P22H5 phage with Se Δ*res* (MOI = 1) under strict anoxic conditions and found that active phage led to substantial bacterial killing compared to heat-inactivated phage (Fig.  1d). This MOI and time was chosen based on growth curves showing maximal bacterial reduction (Fig. S2). We then measured B12 concentrations in the culture supernatant and cell pellet and found that active phage led to substantially increased supernatant B12 with no change in the pellet (Fig.  1e). The peak in extracellular B12 concentration at 5 h coincides with a maximal drop in bacterial density (Fig. S2) and the decrease in B12 by 24 h is consistent with uptake by the surviving bacteria. Next, we evaluated whether a similar effect is observed when the Se-Producer does not produce the complete B12 compound (Se Δ*res* Δ*cbiB*) and found that active phage had a similar antibacterial effect (Fig.  1f) but both the supernatant and pellet contained comparatively trace levels of B12 (Fig.  1g).

### Phage-mediated externalization of bioavailable B12 supports bacterial user growth

**Figure 2:**
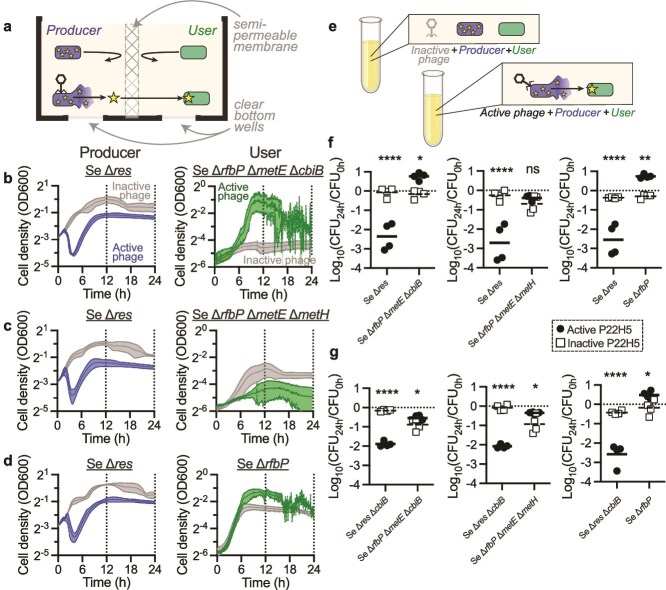
Phage-mediated bacterial lysis enables B12 exchange from Se-Producers to Se-Users. (a) Schematic representation of the dual-culture system (“Duet”) that sequesters bacteria to separate wells but allows metabolite diffusion. (b) In the Duet system, the Se-Producer Se Δ*res* was cultured at an initial density of 0.5 with active or heat-inactive P22H5 at an MOI of 1 and various Se-Users at an initial density of 0.05. Growth of each strain was measured at 600 nm over time by absorbance. Se-User strains included Se Δ*rfbP* Δ*metE* Δ*cbiB*, (c) Se Δ*rfbP* Δ*metE* Δ*metH*, and (d) Se Δ*rfbP*. (e) Schematic representation of direct co-cultures, mixing the Se-Producers with P22H5 phage at an MOI of 1 and Se-Users (i.e. same tube). Concentrations of Se-Producers and -Users were obtained by selective plating for colony forming units (CFUs) at 24 h and results were presented as log fold-change in bacterial density normalized by CFUs at 0 h. (f) The log fold-change in bacterial density of Se-Producers Se Δ*res* or (g) Se Δ*res* Δ*cbiB* cultured with Se Δ*rfbP* Δ*metE,* Δ*cbiB*, Se Δ*rfbP* Δ*metE* Δ*metH* or Se Δ*rfbP*. Statistical significance was determined by two-way ANOVA with a Bonferroni multiple comparisons test (^*^, *P <* .05; ^**^, *P <* .005; ^***^, *P <* .0005; ^****^, *P <* .0001).

To monitor how phage can alter the dynamics of B12 exchange between Se-Producers and Se-Users, we used a co-culture system in which two optically clear microtiter wells are connected by a 0.22 μm semi-permeable membrane. This allows us to monitor bacterial growth in a system where nutrients and phages, but not intact bacterial cells, can diffuse between the two connected wells (Fig.  2a). Separate examination of B12 transport from one well to another shows that substantial diffusion of B12 occurs on the timescale of our experiment (Fig. S3). When adding active P22H5 phage, we found that the Se-Producer strain, Se Δ*res*, was consistently reduced regardless of co-cultured Se-User (Fig.  2b–d). In response to the phage-mediated lysis of this Se-Producer bacterium, there was a substantial increase in the concentration of Se-User, Se Δ*rfbP* Δ*metE* Δ*cbiB*, whereas no growth was observed when the inactive phage was added (Fig.  2b). We also validated these measurements with separate OD measurements at 12 h and 24 h to account for potential effects from cell settling in the wells (Fig. S4). When the Se-Producer Se Δ*res* and P22H5 phage were co-cultured with a Se-User that cannot use B12 but can use Met (Se Δ*rfbP* Δ*metE* Δ*metH*), no Se-User growth was observed (Fig.  2c). This result indicates that B12, not Met, drives the growth of the Se-User. By comparison, a Se-User that does not require supplementation with B12 or Met (Se Δ*rfbP*) showed a modest enhancement of growth with the addition of active P22H5 phage (Fig.  2d), which we suspect is due to reduced competition from the lysed Se-Producer.

For more sensitive and robust endpoint measurements of the effect of phage-mediated B12 externalization, we plated the co-cultures of Se-Producers and Se-Users after 24 h (Fig.  2e). We found that active phage significantly reduced concentrations of Se-Producer, Se Δ*res*, compared to inactive phage, which led to a significant increase in the Se-User that requires B12 or Met supplementation, Se Δ*rfbP* Δ*metE* Δ*cbiB*, but not the Se-User that requires Met and cannot use B12, Se Δ*rfbP* Δ*metE* Δ*metH* (Fig.  2f). Furthermore, the concentration of Se-User that does not require B12 or Met supplementation, Se Δ*rfbP*, increases with the addition of active phage. We suspect this is due to reduced competition from the Se-Producer as we observed in Fig.  2d and Fig. S4A. As additional validation that the release of B12 is important, we used Se-Producer, Se Δ*res* Δ*cbiB*, that has an incomplete B12 pathway. Under this condition, the Se-Users that require B12/Met or Met supplementation (Se Δ*rfbP* Δ*metE* Δ*cbiB* and Se Δ*rfbP* Δ*metE* Δ*metH*, respectively) were unable to grow (Fig.  2g). Although the small but significant increase in Se-Users with active phage indicates reduced death compared to conditions with inactive phage, log fold-change values below zero indicate they did not grow.

As evidence that this phage-mediated cell lysis is crucial to enable B12 availability to other bacterial species, we used a different bacterial species, *E. coli* (Ec) and constructed equivalent phenotypic mutants to their Se-User counterparts. Phage-mediated lysis of the Se-Producer Se Δ*res* led to substantial growth in the Ec-User strain that requires either B12 or Met, but not the strain that only uses Met (Ec Δ*metE* and Ec Δ*metE* Δ*metH*), respectively) (Fig. S5). The Ec-User strain that does not require supplementation with either B12 or Met (Ec Δ*ars*) had an increased growth due to active phage. The nonessential gene *arsB* (an arsenite transporter) was targeted to insert a kanamycin resistance cassette to enable the selective quantification of Ec-User strain (Table 1) [66]. These similar results obtained with Se- and Ec-Users suggest that the phage-mediated lysis mechanism of B12 release may be a generalizable phenomenon to support bacterial growth of other species.

In addition to our expected findings that the phage-mediated release of B12 from Se-Producers is critical to support the growth of Se- and Ec-Users that require B12 for growth, we found that the User strains that did not require B12 supplementation (Se Δ*rfbP* and Ec Δ*ars*) had increased growth due to active phage. This indicates that the antibacterial effect of phage is relieving a competition imposed on the Se-User, which suggests that a relief of competition may also support the growth of Se- and Ec-Users that require B12/Met supplementation. Therefore, in a separate experiment we cultured the Se-Producers that can or cannot produce a complete B12 (Se Δ*res* and Se Δ*rfbP* Δ*metE* Δ*cbiB*, respectively) and collected the sterile-filtered supernatant of their intact cultures (i.e. no phage added) or the phage lysates (i.e. phage added to the culture). We found that when compared to M9 medium, only the phage lysate of Se Δ*res* could support an increased growth in the Se-User strain that requires B12 or Met supplementation (Se Δ*rfbP* Δ*metE* Δ*cbiB*) whereas the Se Δ*res* supernatant of intact cultures had no effect. Additionally, the supernatant or phage lysate of the Se-Producer unable to synthesize a complete B12, Se Δ*res* Δ*cbiB*, did not support growth of the Se-Users (Fig. S6A). Similar results were observed with Ec-Users (Fig. S6B). These results are consistent with our observations in Fig.  2 and indicate that the phage-mediated lysis of Se-Producers is the driving force promoting B12-dependent Users growth.

Our results support several important conclusions. First, the culture supernatant of intact strains that can produce B12 does not contain sufficient B12 to support growth of strains that require its supplementation for growth. By contrast, the phage-mediated lysis of these producers can externalize the necessary amount of B12. Second, the amounts of Met released by phage-mediated lysis are not sufficient to support growth of User strains. Third, although the antibacterial effect of phages may reduce competition from the Producer strains, the externalization of B12 by phage-mediated lysis is the primary driver of growth among User strains. Fourth, the phage-mediated externalization of B12 can facilitate a similar growth of another bacterial species, Ec. Collectively, this suggests that the phage-mediated lysis of one species can increase the extracellular abundance of B12 to support the growth of other species within a consortium.

### Growth of human gut bacteria can depend on B12

**Figure 3:**
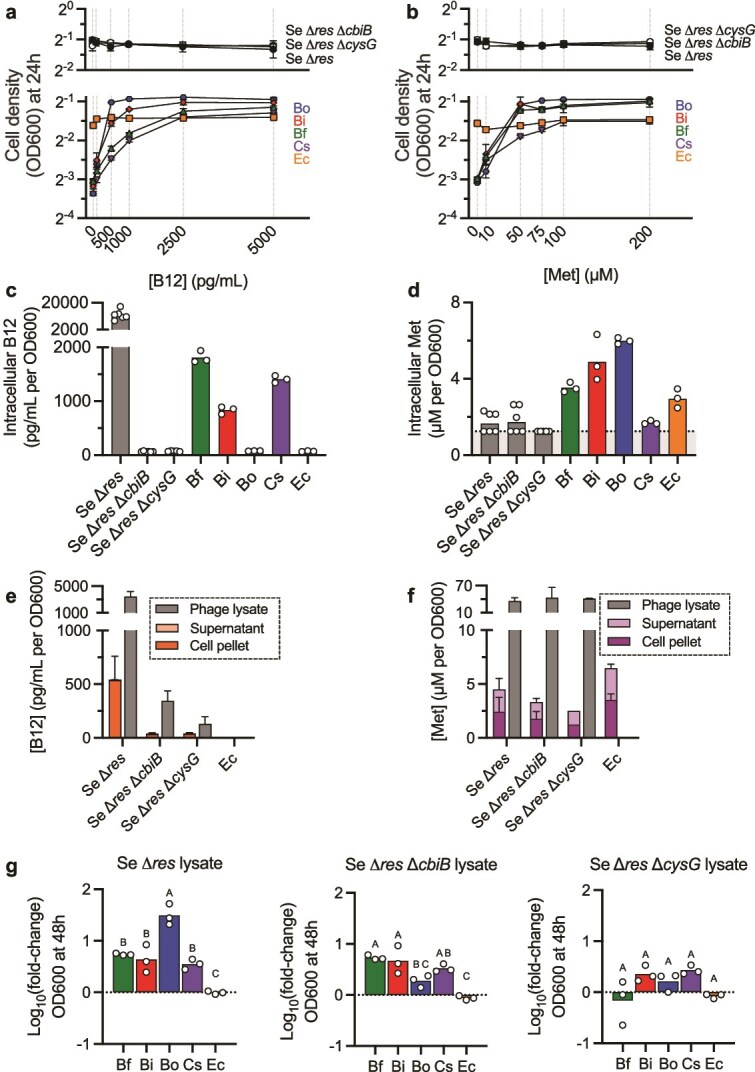
Commensal gut bacteria that must acquire B12 can utilize B12 derived from phage lysates of Se. (a) Bacterial density of Se-Producers and human commensals in DDB medium supplemented with B12 or (b) Met, cultured under anoxic conditions for 24 h at 37°C. (c) Se-Producers and commensal gut bacteria were cultured under anoxic conditions in BHIS medium for 24 h and thrice washed in saline solution before mechanical lysis and measurement of intracellular B12 or (d) Met. (e) The amounts of B12 and (f) Met (methionine) were measured from the supernatant and cell pellets of exponential-phase cultures of Se-Producers grown in unsupplemented DDB medium compared to P22H5 phage lysate cultures with an MOI of 1 for 5 h. (g) The growth of each commensal gut bacteria was measured after 48 h under anoxic monoculture at 37°C using either DDB medium or P22H5 phage lysates of Se-Producer strains grown in DDB medium. The fold-change is calculated by the ratio of growth in phage lysates divided by growth in DDB medium.

Although more than four-fifths of human gut bacteria utilize B12 for metabolic processes including methionine biosynthesis, only a quarter of gut bacteria possess complete B12 biosynthesis pathways [42, 44] supporting that B12 acquisition from the environment is a widespread phenomenon. Our finding that the release of bacterial B12 by phage-mediated cell lysis enables the growth of Se- and Ec-User strains that must acquire B12 suggests that phages may also indirectly modulate the composition of a bacterial community through trophic interactions. To explore this effect, we selected a panel of human gut bacteria for co-culture with our Se-Producer strains. We then formulated a Defined Deficient of B12 (DDB) medium that contains buffer, salts, vitamins, minerals, and amino acids except B12 and methionine. We found that *Bacteroides ovatus* (Bo), *B. intestinalis* (Bi), *B. fragilis* (Bf), and *Clostridium sporogenes* (Cs) cultured in DDB medium require B12 supplementation for growth, whereas *E. coli* (Ec) does not require it for growth (Fig.  3a). We chose these species because they are prevalent components of the human gut [67–69], have predicted growth dependencies on B12 [36, 70] that we validate here, can all grow in our DDB medium, and we are able to distinguish each species by 16S rRNA amplicon sequencing [71, 72]. Additionally, the growth of Se-Producers in this medium did not require B12 supplementation (Fig.  3a). As an additional control to our Se Δ*res* and Se Δ*res* Δ*cbiB* strains, we generated a new Se-Producer strain with a *cysG* gene deletion that disables the first biosynthetically dedicated step in the B12 pathway [73]. This strain, Se Δ*res* Δ*cysG*, is unable to produce B12 or its precursors including AdoCby (Fig.  1a, Table 1). We found that it similarly does not require B12 supplementation for growth (Fig.  3a). When investigating the effect of Met supplementation on growth, we similarly found that Se-Producers and Ec did not require Met for growth, whereas the other bacteria (Bo, Bi, Bf, and Cs) could utilize Met, requiring it at substantially higher concentrations than B12 (Fig.  3b). To simplify the later discussion, we describe the commensal bacteria (Bo, Bi, Bf, and Cs) that must acquire B12 or Met for growth under our defined conditions as Commensal-Users, which does not include Ec because it can grow regardless of B12/Met supplementation.

We next determined the extent of B12 and Met retained intracellularly during bacterial growth across these different species. We grew bacteria in BHIS, which is an undefined rich medium that contains B12 and is commonly used to grow bacteria with unknown or complex nutrient requirements, including gut anaerobes [74]. Se Δ*res* contained far greater intracellular concentrations of B12 than any other bacterium including the other Se-Producers that are deficient in B12 biosynthesis (Fig.  3c). Among the Commensal-Users (Bo, Bi, Bf, and Cs), all contained significant quantities of intracellular B12 imported from BHIS medium, except for Bo. This suggests that Bo’s growth may not solely depend on B12 when excess Met is present in this rich medium. When examining intracellular Met concentrations, all strains analyzed contained intracellular methionine, though all were <8 μM per OD600 (Fig.  3d). Met is an essential amino acid and these varying levels reflect only the free amino acid released; it is possible that proteinaceous methionine is present in abundance. Additionally, Bo contained relatively high amounts of Met, supporting non-B12 dependent mechanisms of Met acquisition.

### Phage lysed Se releases physiologically relevant quantities of intracellular B12

To evaluate whether substantial quantities of B12 can be externalized by phage-induced cell lysis, we measured the intracellular and extracellular concentrations of B12 from intact exponential phase Se-Producer cultures and compared it to the amount released to the extracellular environment after lysis by P22H5 phage. The intact Se-Producer Se Δ*res* maintains B12 intracellularly within the cell pellet with negligible amounts in the supernatant whereas phage-mediated lysis of bacterial cells releases considerable amounts to the extracellular environment (“Phage Lysate”) (Fig.  3e). Also, in line with expectations, comparatively little B12 was found in the Se-Producers that are unable to produce a complete B12 (e.g. Se Δ*res* Δ*cbiB* and Se Δ*res* Δ*cysG*) or in Ec. When analyzing Met in a similar manner (Fig.  3f), we found that the intracellular/extracellular distribution of Met for intact cells remained similar across the Se-Producers and Ec, in addition to the amounts released from Se-Producers with phage. We found greater amounts of B12 and Met in the phage lysates compared to the cultures of intact cells, which is likely due to the extracellular accumulation of B12 and Met during the 5 h culture. By contrast, these concentrations would be regulated intracellularly. These data confirm that substantial quantities of B12 are released by phage-mediated cell lysis.

We next determined the extent of growth for each commensal bacterium supported by B12 produced from phage-mediated lysis of Se-Producers. The phage lysates of Se Δ*res* led to significantly increased growth for each Commensal-User (Bf, Bi, Bo, and Cs) compared to Ec, which does not require B12 supplementation (Fig.  3g). Furthermore, the phage lysates of Se Δ*res* Δ*cbiB* could support the significant growth of Bf, Bi, and Cs, but not Bo, whereas the phage lysates of Se Δ*res* Δ*cysG* had minimal effect. These results suggest that Bo only utilizes the complete B12 produced by Se (pseudo-B12) whereas Bf, Bi, and Cs can utilize both complete and incomplete B12 (AdoCby). The differential capacity of these Commensal-User strains to utilize Se-derived pseudo-B12, or its precursors, including the possibility of cobamide precursor salvaging, is consistent with previous observations that cobamide preferences vary widely across bacteria [75]. Next, to evaluate whether phage-mediated lysis of Se-Producers were necessary to support the growth of commensals, we prepared DDB medium supplemented with the supernatants of intact Se-Producer cultures (i.e. no phage added). We found that none of the Se-Producer supernatants could promote growth of Bo, Bi, or Bf (Fig. S7), which is consistent with our previous results showing that Se Δ*res* supernatants did not contain significant levels of B12 (Fig.  3e and Fig. S6). Additionally, the supernatants of cultures of all intact Se-Producer supported partial growth of Cs to levels comparable to methionine supplementation. We speculate that this might be due to the ability for *Firmicutes* like Cs to derive amino acids from extracellular proteins [76]. No significant effect on Ec growth was observed. Collectively, these results show that phage-mediated lysis of a Se-Producer can release B12 to support the growth of commensal bacteria that must acquire it for growth.

### Phage-mediated lysis of Se alters a bacterial consortium in a B12-dependent manner

**Figure 4:**
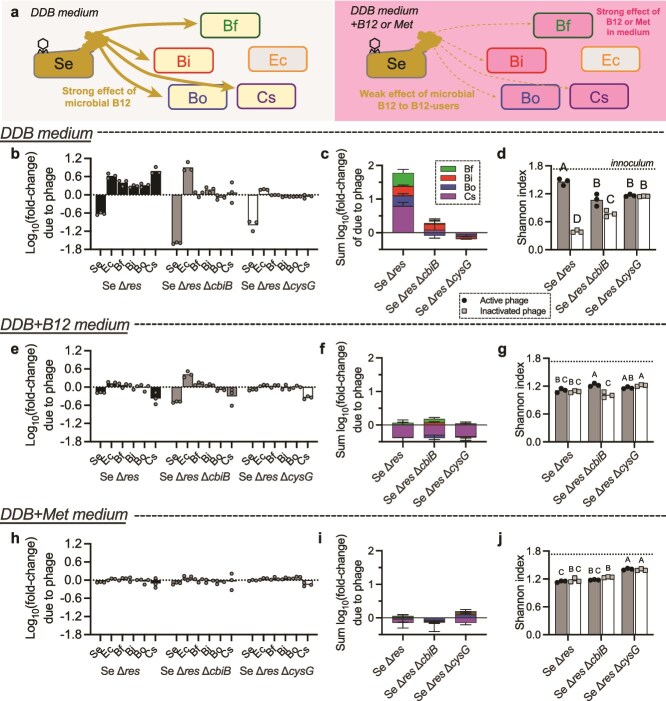
Phage-mediated lysis of a B12-Producer alters the composition of a bacterial community in a B12-dependent manner. (a) Schematic representation of the co-culture experiments showing that the addition of phage to lyse the producer, Se, can release intracellular B12 to support the growth of B12-users and that the magnitude of the effect caused by phage depends on whether significant quantities of B12 or Met are present in the environment. (b) The log fold-change in concentration of each bacterium due to active versus heat-inactivated P22H5 phage in co-cultures of Se, Ec, Bf, Bi, Bo and Cs. Cultures inoculated at an initial density of 0.01 were grown under anoxic conditions at 37°C for 24 h. Phages were inoculated at an MOI of 0.0001. (c) The sum of log fold-change of the Commensal-Users (Bo, Bi, Bf, and Cs). (d) The Shannon Index of the community. The dashed horizontal line represents the Shannon Index of the inoculum. (e) The log fold-change of bacteria concentration due to phage, (f) sum of the log fold-change of the Commensal-Users, and (g) Shannon Index of the same culture in DDB medium supplemented with 5 ng/ml of B12 or (h,i,j) 100 μM of Met. Statistical significance was determined by two-way ANOVA or a mixed-effects model with a Bonferroni multiple comparisons test. Unique letter codes indicate statistical significance with *P <* .05.

With our data showing that B12 is released from Se-Producers by phage-mediated lysis and that this B12 can support the growth of various human gut bacteria, we tested our hypothesis that the phage-mediated lysis of a B12-Producer in a mixed bacterial community could substantially modulate the community composition by B12 externalization. We first confirmed that various combinations of the Commensal-Users (Bo, Bi, Bf, and Cs) did not lead to bacterial growth in unsupplemented DDB medium (Fig. S8). Next, we co-cultured the entire community (Bo, Bi, Bf, Cs, and Ec) with a Se-Producer and its cognate P22H5 phage for 24 h. We used DDB medium to evaluate how B12 externalized by phage-mediated lysis would affect the consortium (Fig.  4a). In unsupplemented medium, we expect that B12 externalized by lysis of the Se-Producer will support the growth of Bf, Bi, Bo, and Cs whereas medium supplemented with B12 or Met will allow these strains to grow independently of the Se-Producer derived B12, muting the effect of phage.

In co-cultures of the entire community, we calculated the log fold-change of each bacterial species due to active phage compared to inactive phage (Fig.  4b). The introduction of active P22H5 phage led to the expected reduction in Se Δ*res* concentration compared to inactive phage, which coincided with increases in all other bacterial species. When Se-Producer strains that produce incomplete B12 or no B12 (Se Δ*res* Δ*cbiB* or Se Δ*res* Δ*cysG*, respectively) were lysed by phage, only Ec had a measurable increase. The sum of fold-change in the absolute abundance of the commensals that must acquire B12 was substantially higher with Se Δ*res* compared to Se Δ*res* Δ*cbiB* or Se Δ*res* Δ*cysG* (Fig.  4c). Furthermore, the phage-mediated lysis of Se Δ*res* significantly increased the Shannon Index of the community by 3.73-fold whereas lysis of Se Δ*res* Δ*cbiB* or Se Δ*res* Δ*cysG* had a comparatively minimal effects of 1.38-fold and 1.01-fold, respectively (Fig.  4d). Additionally, the Shannon Index of Se Δ*res* Δ*cbiB* and Se Δ*res* Δ*cysG* mixed community cultures with inactive phage were higher than the culture of Se Δ*res* with inactive phage. It is likely that these Se-Producer mutants that cannot form pseudo-B12 are less competitive because they must rely on the B12-independent methionine synthase encoded by *metE*, which has considerably slower turnover than the B12-dependent methionine synthase encoded by *metH* [77]. Overall, our results using DDB medium indicate that phages can indirectly modulate the composition of a microbial community *via* externalization of B12 in a manner independent of the phage’s reduction of bacterial concentration (i.e., the antibacterial effect).

We next evaluated whether supplementation of the medium with B12 or Met could alter the effects induced by phage-mediated lysis of the Se-Producers. In contrast to our observations with unsupplemented medium, the inclusion of B12 or Met muted the phage effect on the concentrations of the commensals (Fig.  4e and h, respectively), the Commensal-Users that must acquire B12 (Fig.  4f and i, respectively), and the Shannon Index (Fig.  4g and j, respectively).

We validated that active phage propagation was occurring in these co-cultures by measuring the phage concentrations and found that they increased substantially from the inoculated concentration of 5 × 10^2^ pfu/ml to reach final concentrations ranging between 10^6^ to 10^10^ pfu/ml, with comparatively lower concentrations when the medium was supplemented with B12 or Met (Fig. S9). The reduced phage titers correspond with reduced effects of active phage on the Se-Producers compared to unsupplemented medium (Fig.  4b, e, and h). We speculate that the B12 and Met supplementation increases the competitiveness and growth of the Commensal-Users (Bo, Bi, Bf, and Cs) which then reduces the growth of Se-Producers and consequently the productivity of phage predation. For co-cultures where Se Δ*res* was the Se-Producer, roughly 40%–60% were phage resistant bacteria regardless of medium supplementation (Fig. S10). Although bacterial monocultures typically become completely phage resistant, the introduction of even one other species can provide sufficient competition that the fitness cost of phage resistance is less favorable than remaining phage susceptible [78]. We then evaluated whether a long-term coexistence between P22H5 and Se Δ*res* or Se Δ*res* Δ*cysG* could be maintained within the bacterial consortium. With daily serial passage via 100-fold dilutions into fresh DDB medium, we found that Se-Producers persisted though with comparatively lower levels with active phage versus inactive phage (Fig. S11A) and phage concentrations were also maintained (Fig. S11B). Furthermore, the frequency of phage resistance for Se-Producers peaked within the first 24 h and then declined in the remaining time points (Fig. S11C). Collectively this suggests that phages and their bacterial hosts can coexist within mixed microbial communities for long periods, maintaining the B12 externalization mediated by phage.

### Additional environmental perturbations can modulate phage-driven effects

A microbial community in a natural environment is likely to encounter several avenues of perturbation that can be confounding. Therefore, we built on our findings by increasing the complexity of our consortia and evaluated the significance of phage-mediated B12 release within two specific contexts of perturbation: the targeted knockdown of another bacterium within the consortium by phage and a broad antimicrobial activity mediated by an antibiotic.

**Figure 5:**
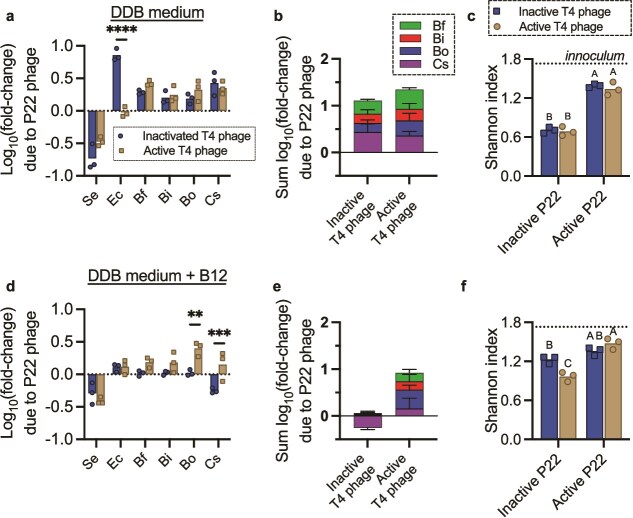
*Escherichia coli* the phage T4 reveals P22-driven community alteration in B12-supplemented medium. Mixed-culture experiments were performed as described in Fig.  4, with addition of *E. coli* phage T4. Ec, Bf, Bi, Bo, Cs and the Se-Producer strain (Se Δ*res*) were cultured at an initial density of 0.01 at 37°C for 24 h in DDB medium with or without B12 supplementation. Phage P22H5 was inoculated at an MOI of 0.0001 and phage T4 at an MOI of 0.01. (a) The log fold-change in bacterial concentration due to active versus heat-inactivated P22H5 phage, when exposed to inactive or active T4 phages. (b) The sum of log fold-change of the Commensal-Users due to P22H5 with inactive or active T4 phages. (c) The Shannon Index of the community. The dashed horizontal line represents the Shannon Index of the inoculum. (d–f) Corresponding analyses were performed on the same culture in DDB medium supplemented with 5 ng/ml of B12. Statistical significance was determined by two-way ANOVA or a mixed-effects model with a Bonferroni multiple comparisons test. Unique letter codes indicate statistical significance with *P <* .05.

Using the same experimental conditions as described for Fig.  4, we included an additional phage, *E. coli* phage T4. We found that the inclusion of inactivated T4 phage in these experiments (Fig.  5a) replicated our prior result showing that P22H5 phage lysis of Se Δ*res* leads to an enrichment in the Commensal-Users and Ec (Fig.  4b). In contrast, active T4 phage inhibited the growth enrichment of Ec whereas there was no significant impact on the Commensal-Users (Fig.  5a and b) nor an impact on the community diversity (Fig.  5c). When including B12 and inactivated T4 phage, much of the effects from P22H5 phage were muted (Fig.  5d) as we observed previously (Fig.  4e). However, when B12 and active T4 phage were present, concentrations of all Commensal-Users were increased, though only Bo and Cs significantly so (Fig.  5d), which had an increased sum fold-change among Commensal-Users (Fig.  5e). Consistently, active P22H5 phage led to the greatest diversity (Fig.  5f). We validated that, when added, P22H5 and T4 phage propagation was occurring in these co-cultures by measuring the phage concentrations (Fig. S12). Considering that Ec does not retain B12 (Fig.  3e) and does not benefit directly from the phage-mediated externalization of B12 from Se Δ*res* (Fig.  3g), our data show that in DDB medium the impact of T4 phage is limited to the Ec-Se pairing and does not significantly affect the growth of Commensal-Users that rely on Se Δ*res* lysis. However, when B12 is supplemented in the medium, the effect of P22H5 phage is revealed when T4 phage suppresses Ec enrichment. These data indicate that in addition to the impact of P22H5 phage to modulate the bacterial consortium through B12 release, an additional phage such as T4 phage can impose further alterations with an extent that is dependent on other phages and nutrient supplementation in the environment.

To evaluate the extent to which antibiotics could have on phage-mediated modulation of bacterial community composition, we first titrated the concentration of ampicillin on each member of the consortium. We found that 5 and 50 μg/ml had varying impact on Se and Ec, consistently killed Cs, but did not affect Bf, Bi or Bo (Fig. S13). When these concentrations of ampicillin were included in the community co-culture, we found that P22H5 phage had no impact on Se Δ*res* nor on the consortium. Higher doses of ampicillin reduced diversity (Fig. S14A–D). Similar effects were observed when B12 was supplemented in the medium (Fig. S14E–H). These data suggest that when an additional insult is present, the effect of phage-mediated lysis can be muted, disrupting community interactions.

## Discussion

In this study, we reveal a mechanism by which phages can mediate the externalization of a metabolically valuable compound that is typically retained intracellularly—vitamin B12. We first use genetically well-defined mutants of Se, one capable of producing B12 and another that must acquire B12 or Met from the environment, to show that Se-Producer lysis by P22H5 is essential to externalize sufficient amounts of B12 to support the growth of a Se-User in co-culture. Additionally, other intracellular materials released from the Se-Producer (i.e., methionine) were unable to stimulate growth in the Se-User. To demonstrate that phage-mediated cell lysis and release of B12 has the potential to fundamentally modulate microbial communities, we co-cultured the Se-Producer with a consortium of human gut bacteria that must acquire B12 from the environment. We showed that the addition of lytic phage leads to substantial increases in the concentrations of these Commensal-Users, leading to an increased diversity, i.e., not achieved by the supplementation of B12 or methionine in the medium.

Our work details a mechanism by which typically intracellular metabolites can become available extracellularly in naturally occurring microbial communities. Studies of naturally occurring microbial communities have shown that the exchange of microbial metabolites is an important contributor to community stability and diversity [21], with the mechanisms of metabolite acquisition an area of active interest [27]. Equally if not more interesting is the how and why metabolically valuable microbial metabolites are made available for use by other species. Some molecules like gases and small hydrophobic molecules can permeate cell membranes, whereas other molecules that are larger and charged require transport systems [79]. For example, exporters are known for several amino acids including alanine [80], arginine [81], homoserine [82], leucine [83], phenylalanine [84], and threonine [82] in *E. coli*, in addition to ammonium in *Rhodopseudomonas palustris* [85]. Although mutations may be needed to increase export or reduce their recapture, these known excretion mechanisms can explain how certain metabolites can be exchanged in co-cultures including those based on methionine, arginine, and acetate [86]; methionine, histidine, arginine, and tryptophan [87]; several amino acids [24]; and ammonium [88]. Although it is well appreciated that B12 acquisition from the environment occurs, the mechanisms by which B12 is externalized from bacteria remain unknown [89]. Prior work has shown that the debris produced by phage-mediated lysis of Ec can support the growth of co-cultured Se [90], and that the sterile phage lysates of non-auxotrophic Ec and *Bacteroides thetaiotaomicron* can support the growth of amino acid auxotrophs of Ec [29]. Our work significantly advances these lines of inquiry by showing that cell lysis by phage releases substantial quantities of B12 that are otherwise sequestered intracellularly. In contrast to amino acids, vitamin B12 is needed in very small quantities but requires extensive resources for its biosynthesis. This explains why B12-utilizing bacteria benefit from acquiring B12 from the environment, but does not explain why B12-producing bacteria would allow it to be available extracellularly. Our work reconciles this inconsistency, showing that phage-mediated lysis can facilitate the release of intracellular B12 at physiologically relevant concentrations.

From a broader community perspective, our work shows that additional mechanisms of bacterial interaction can be mediated by the externalization of nutrients that results from phage-mediated cell lysis. Separate from how intact bacteria can cooperate or compete, it is the detritus of lysed cells that provides the benefit, which can be an often unaccounted factor as a bacterial nutrient source. Additionally, we found that the greatest community diversity was achieved by phage lysis of the B12 producing Se, and not by exogenous supplementation of B12 or Met, which highlights the importance of this mechanism for community interaction and possibly general host health which is correlated to microbiome diversity [91]. Although there is abundant evidence that diet can alter the bacterial community [92], our work indicates that phages are important for facilitating the recycling of scarce but valuable metabolites.

Although phage-mediated lysis is a major mechanism in naturally occurring microbiomes by which bacteria are lysed to release their intracellular content, it is unlikely to be the only mechanism. High-yield producers such as *Propionibacterium acidipropionici* [93] and various marine bacteria contain B12 in culture supernatants [46], which suggests yet undescribed mechanisms of B12 externalization. Additionally, protists consume bacteria for general nutrients [94] and bacteria can secrete bacteriolytic antibiotics for phosphorous [95]. However, phages are widespread entities that are often found wherever bacteria are present. In marine ecosystems, where phages are estimated to lyse 20%–40% of surface bacteria daily to facilitate the crucial process of nutrient recycling, (i.e. the “viral shunt”) [17, 96], phage-mediated cell lysis plays important roles in how vitamins become available in these nutrient-depleted environments [34], which can significantly alter the microbial composition [32, 97, 98]. With broad compositional changes induced by the externalization of the metabolically valuable compound, B12, our results demonstrate a functionally significant mechanism by which the ongoing phage predation that typically occurs in the environment and the mammalian gut can promote community diversity.

## Supplementary Material

Article_Phage-induced_crossfeeding_v29_supplemental_wrag160

## Data Availability

The 16S rRNA gene amplicon sequencing data are available in National Center for Biotechnology Information BioProject (PRJNA1441165). All the figure datasets are accessible in the Zenodo repository (doi:10.5281/zenodo.20451878).
