# Supplementary material for: Bacteriophage-mediated cell lysis externalizes a metabolically valuable nutrient to broadly modulate bacterial communities
Source: ISME J. 2026 Jun 23;20(1):wrag160. doi: 10.1093/ismejo/wrag160 (PMC13440303; doi:10.1093/ismejo/wrag160)
Supplement: Supplementary file 1 [file article_phage-induced_crossfeeding_v29_supplemental_wrag160.pdf]

Supplementary information for the manuscript:

Bacteriophage-mediated cell lysis externalizes a metabolically valuable nutrient to broadly modulate bacterial communities

David da Silva Barreira,[1] Hannah Poole,[1] Rogério A. Bataglioli,[1] Bryan B. Hsu[1,*]

1. Department of Biological Sciences
   Virginia Tech
   Blacksburg, VA 24061

   Fralin Life Sciences Institute
   Virginia Tech
   Blacksburg, VA 24061

   Center for Emerging Zoonotic, Arthropod-borne Pathogens
   Virginia Tech
   Blacksburg, VA 24061

* Corresponding author: bhsu@vt.edu
Bryan Hsu
Department of Biological Sciences
Virginia Tech
Life Sciences 1 Building
970 Washington St SW
Blacksburg, VA 24061
USA

SUPPLEMENTAL FIGURES

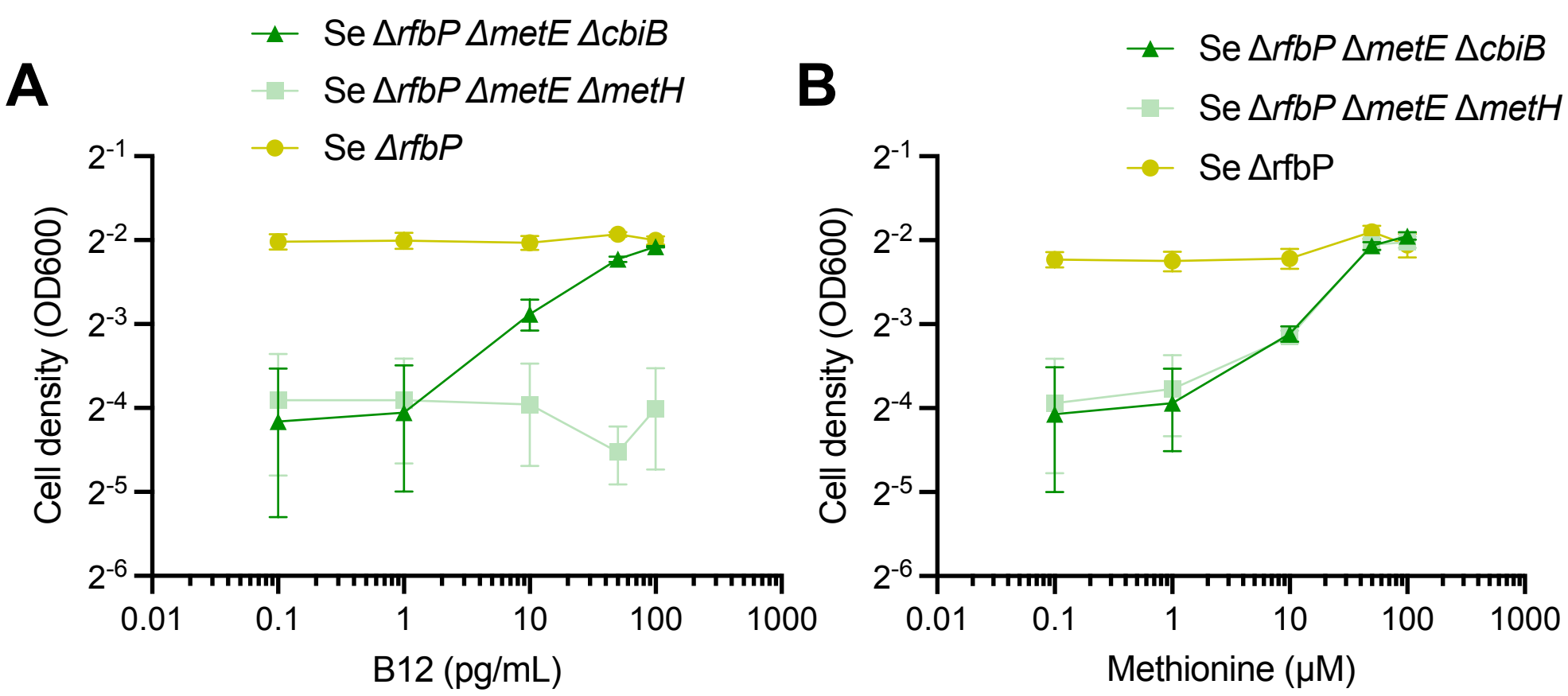

Fig. S1. Cell density of Se-Users after 24 h culture. Se-Users were cultured in M9 minimal media supplemented with **(A)** B12 or **(B)** methionine.

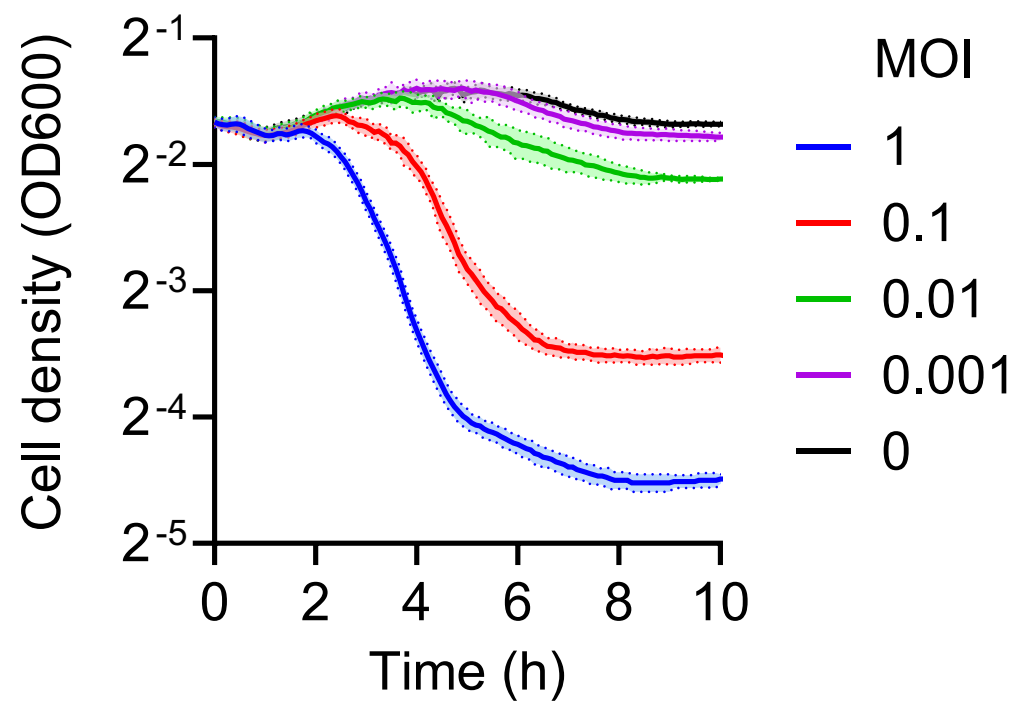

Fig. S2. Growth curves of Se as a function of phage P22H5 MOI.

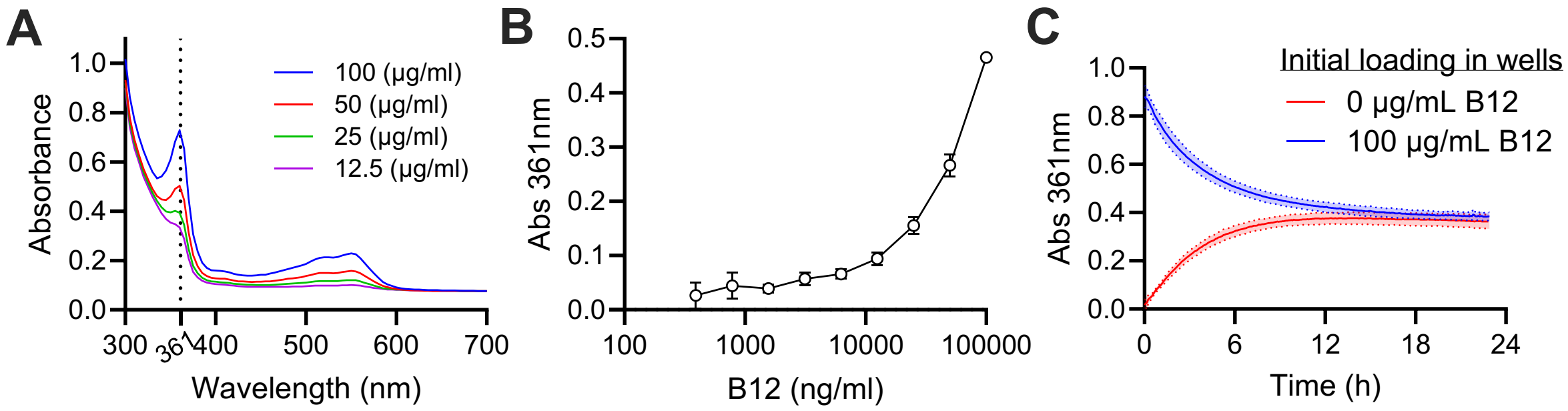

Fig. S3. Diffusion rate of B12 across Duet co-culture wells. (A) B12 has a characteristic absorbance at 361 nm that **(B)** can be measured in a concentration dependent manner. **(C)** Monitoring of B12 absorbance in two wells containing M9 minimal medium, shaken at 37°C, shows B12 loaded into one well can diffuse into the adjoining well to eventually reach equilibrium concentration. Solid line represents the mean and dashed lines the standard deviation.

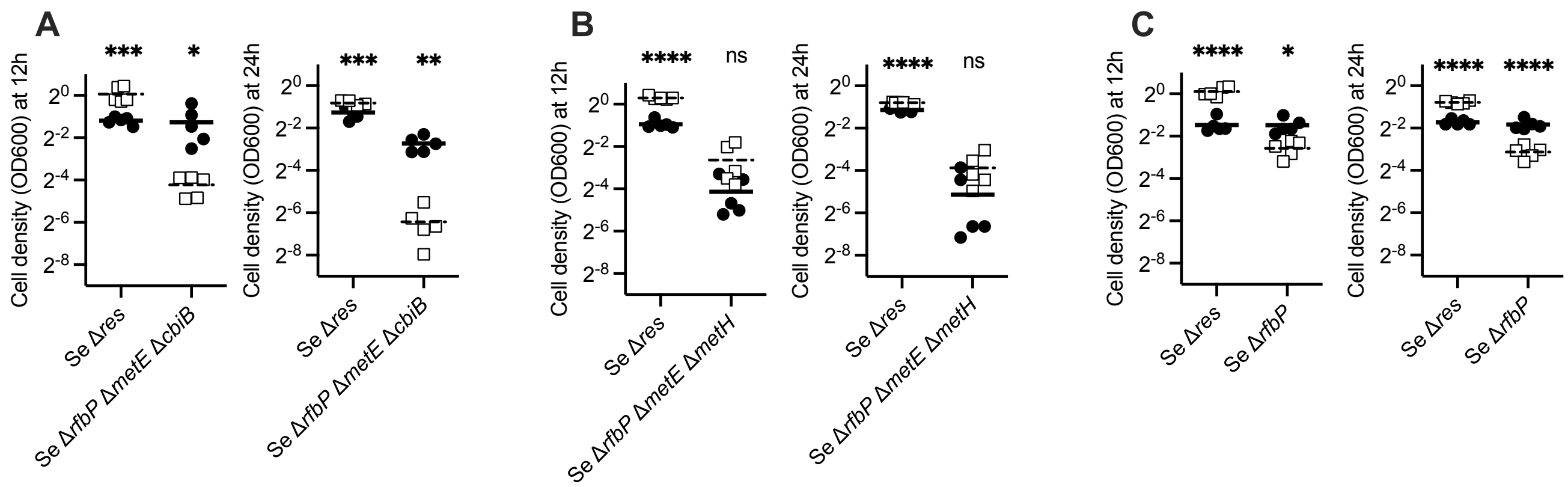

Fig. S4. Measure of bacterial density after complete cell resuspension of co-culture experiments performed with the Duet system. The bacterial density of co-cultures grown with the Duet system under conditions described in Figure 2A-D, were measured by optical density at 600 nm (OD600) at 12 h and 24 h after complete cell resuspension. **(A)** The Producer strain Se Δ*res* was cultured with the User strains Se Δ*rfbP* Δ*metE* Δ*cbiB*, **(B)** Se Δ*rfbP* Δ*metE* Δ*metH*, or **(C)** Se Δ*rfbP*. Statistical significance was determined by two-way ANOVA with a Bonferroni multiple comparisons test (*, $P<0.05$; **, $P<0.005$; ***, $P<0.0005$; ****, $P<0.0001$).

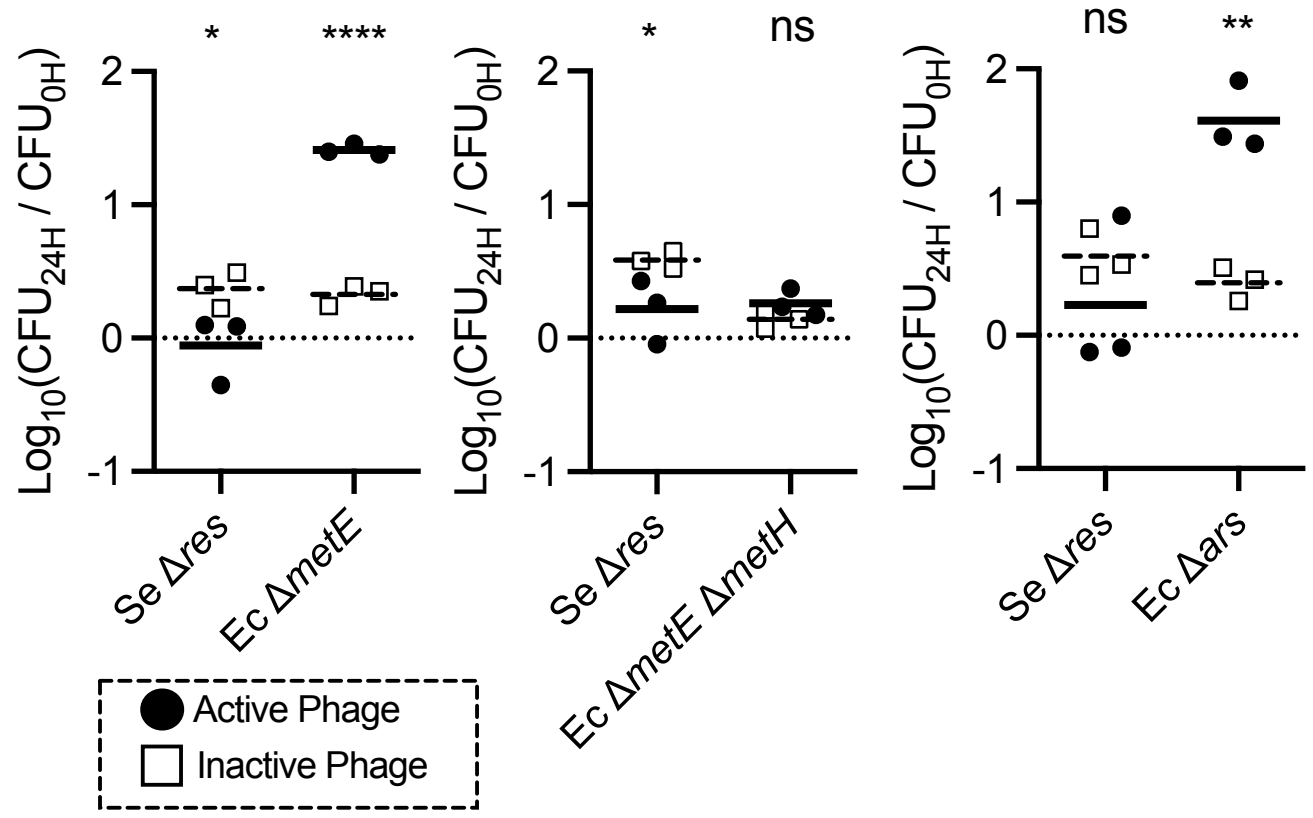

Fig. S5. Phage-induced lysis of Se-Producers enhances interspecies cross-feeding of B12. These results were obtained following the same experimental procedure as described in Figure 2E-G. The log fold-change in bacterial density of Se-Producers Se Δ*res* cultured with Ec Δ*ars*, Ec Δ*metE*, or Ec Δ*metE* Δ*metH*. Statistical significance was determined by two-way ANOVA with a Bonferroni multiple comparisons test (*, P<0.05; **, P<0.005; ***, P<0.0005; ****, P<0.0001).

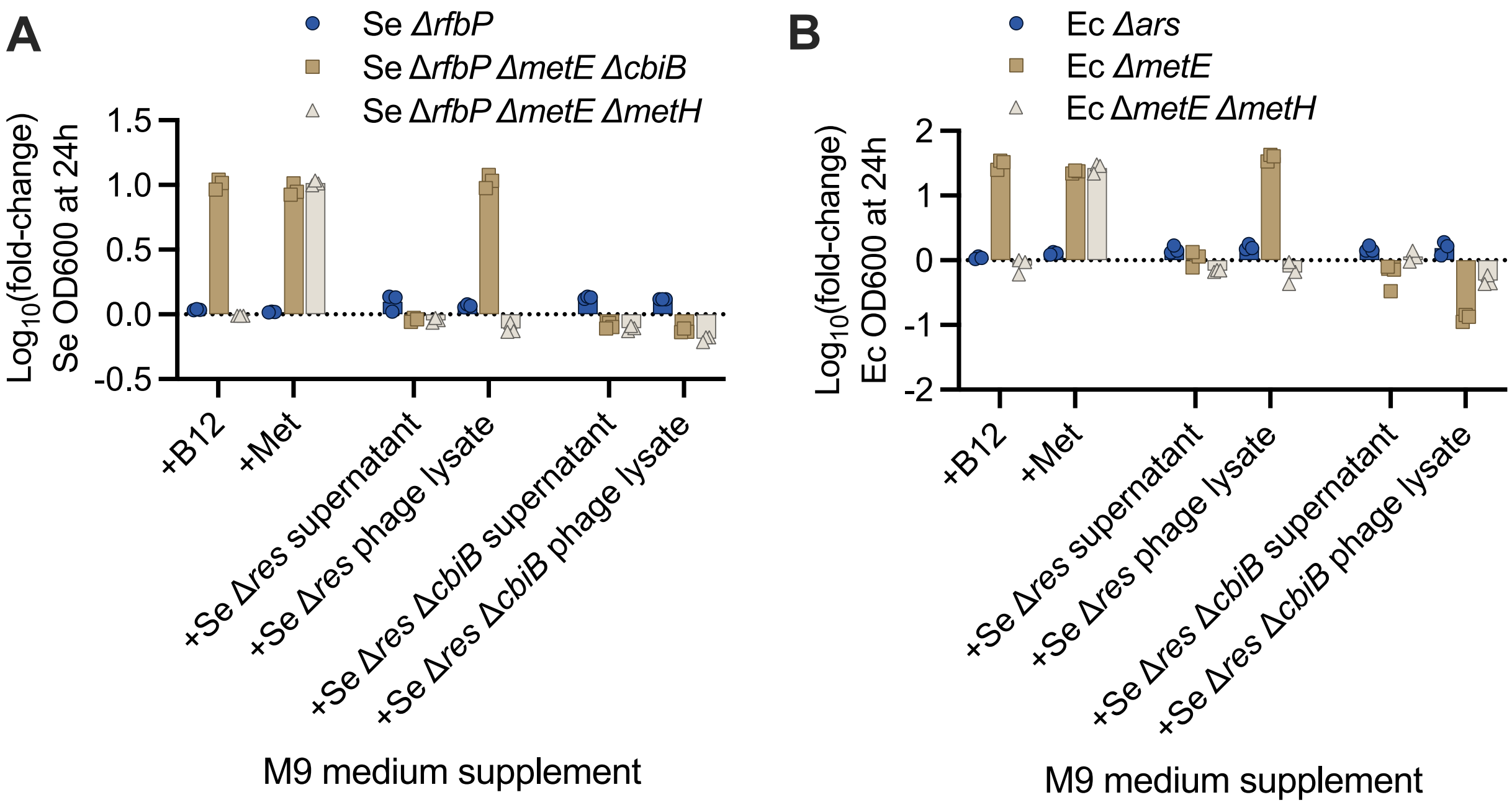

Fig. S6. Growth of Se- and Ec-User strains in M9 medium supplemented with supernatants and phage lysates of Se-Producers. (A) The log fold-change of Se-Users, and **(B)** Ec-Users culture M9 minimal media supplemented with phage lysates and culture supernatants of Se-Producers. Phage lysates and supernatants of Se-Producers were obtained following the experimental conditions described in Figure 2. Fold-change were calculated by the ratio of growth in supernatants normalized to growth without supplementation.

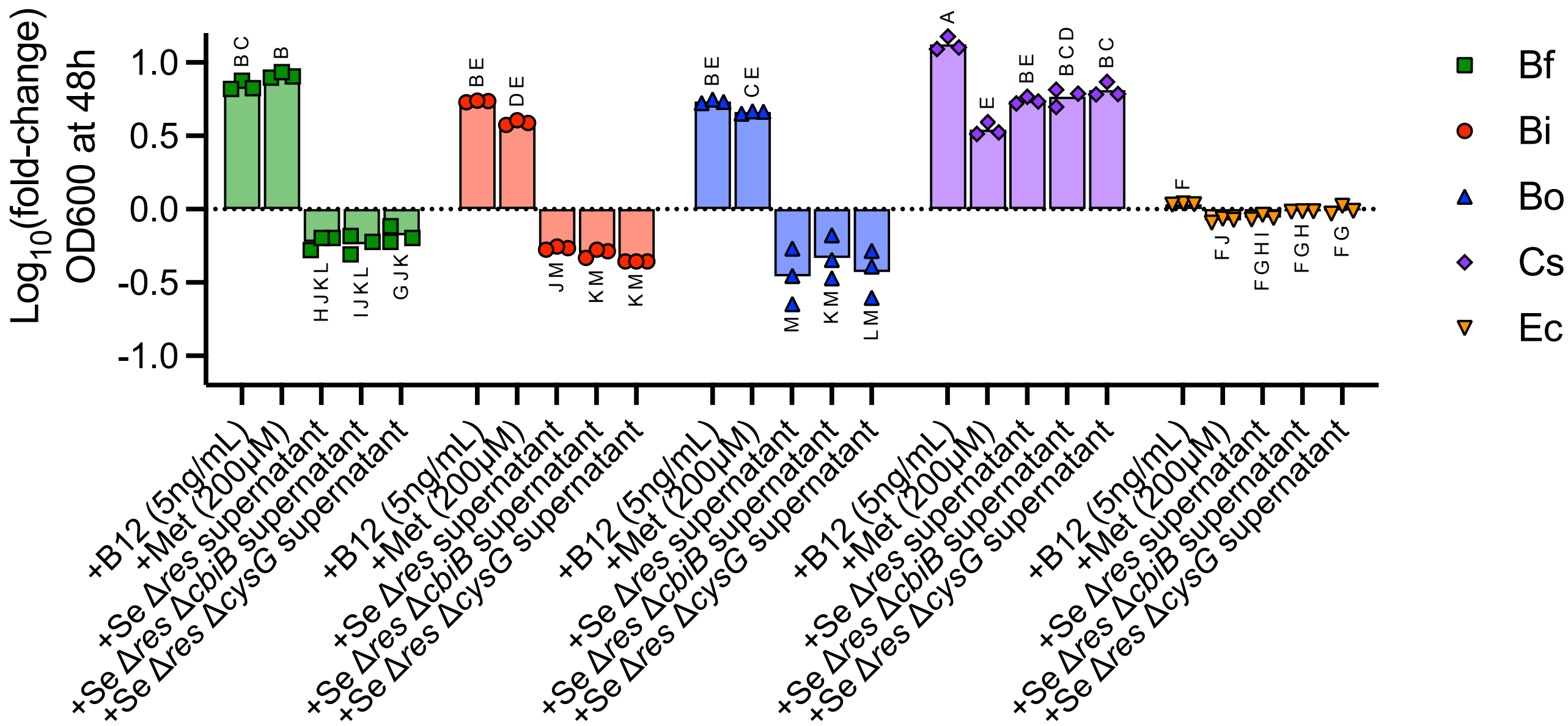

Fig. S7. Growth of User strains in DDB medium supplemented with supernatants of intact cultures of Se-Producers. Users were cultured under anoxic monoculture at 37°C in DDB medium supplemented with supernatants of Se-Producers that did not receive phage. Fold-change were calculated by the ratio of growth in supernatants normalized to growth without supplementation.

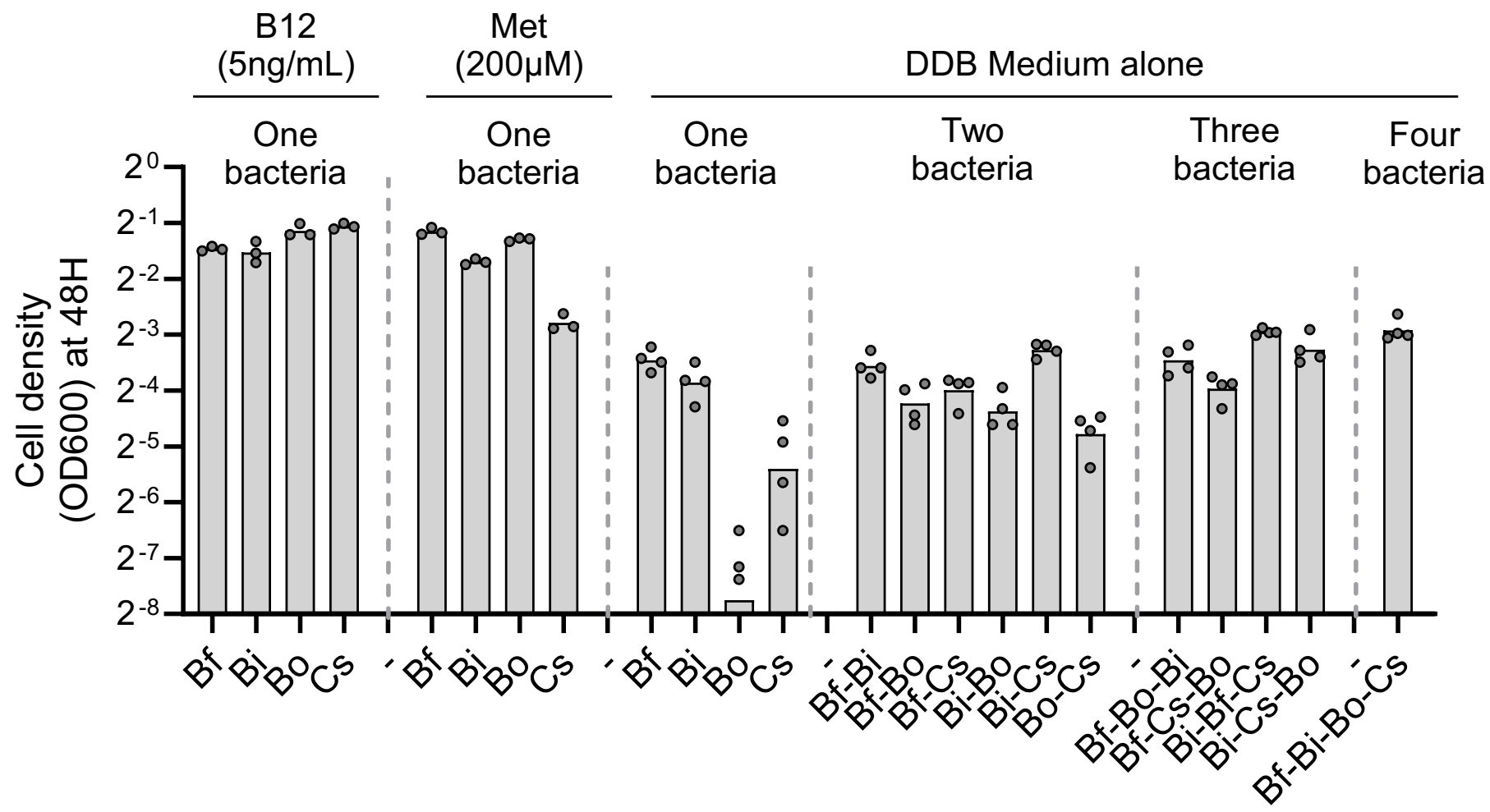

Fig. S8. Growth of User strains alone or in combination in un-supplemented DDB medium.

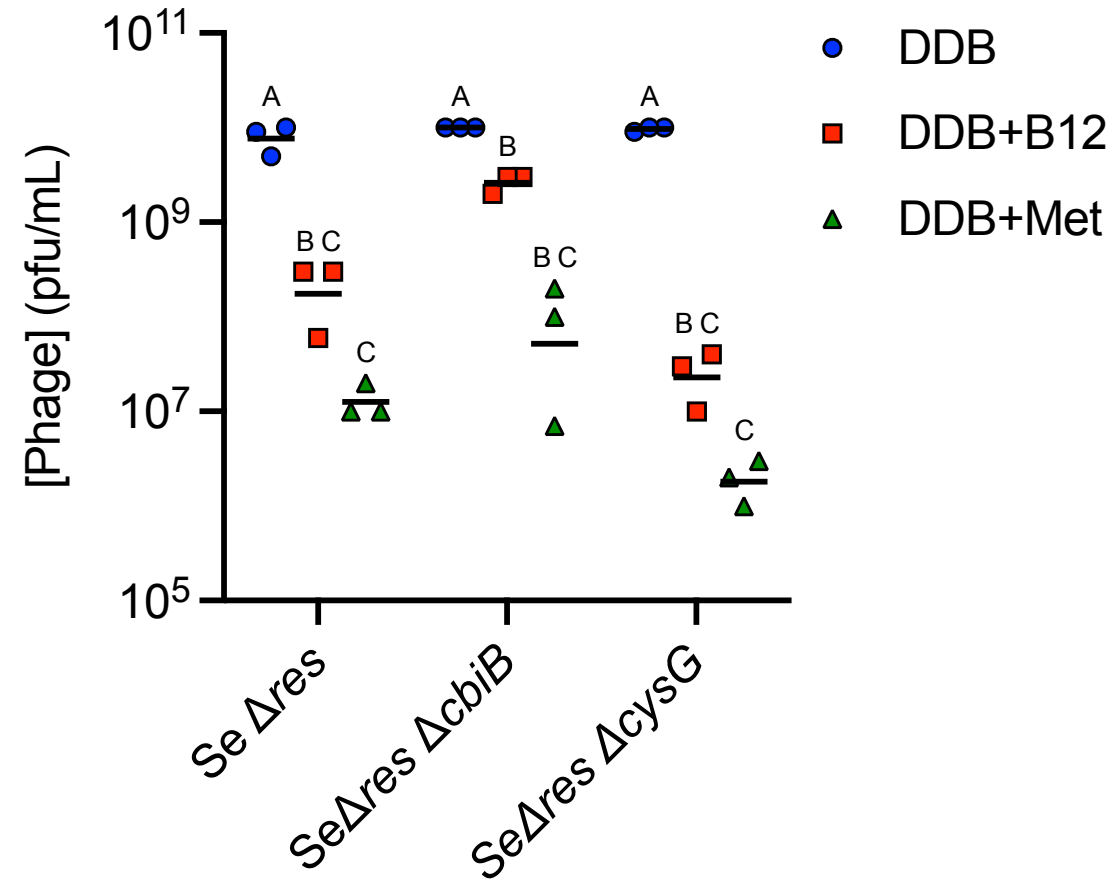

Fig. S9. P22H5 phage titers measured after 24 h in mixed-culture experiments. Statistical analysis by two-way ANOVA with Tukey's multiple comparisons test. Distinct letters indicate comparisons with statistical significance (P<0.05).

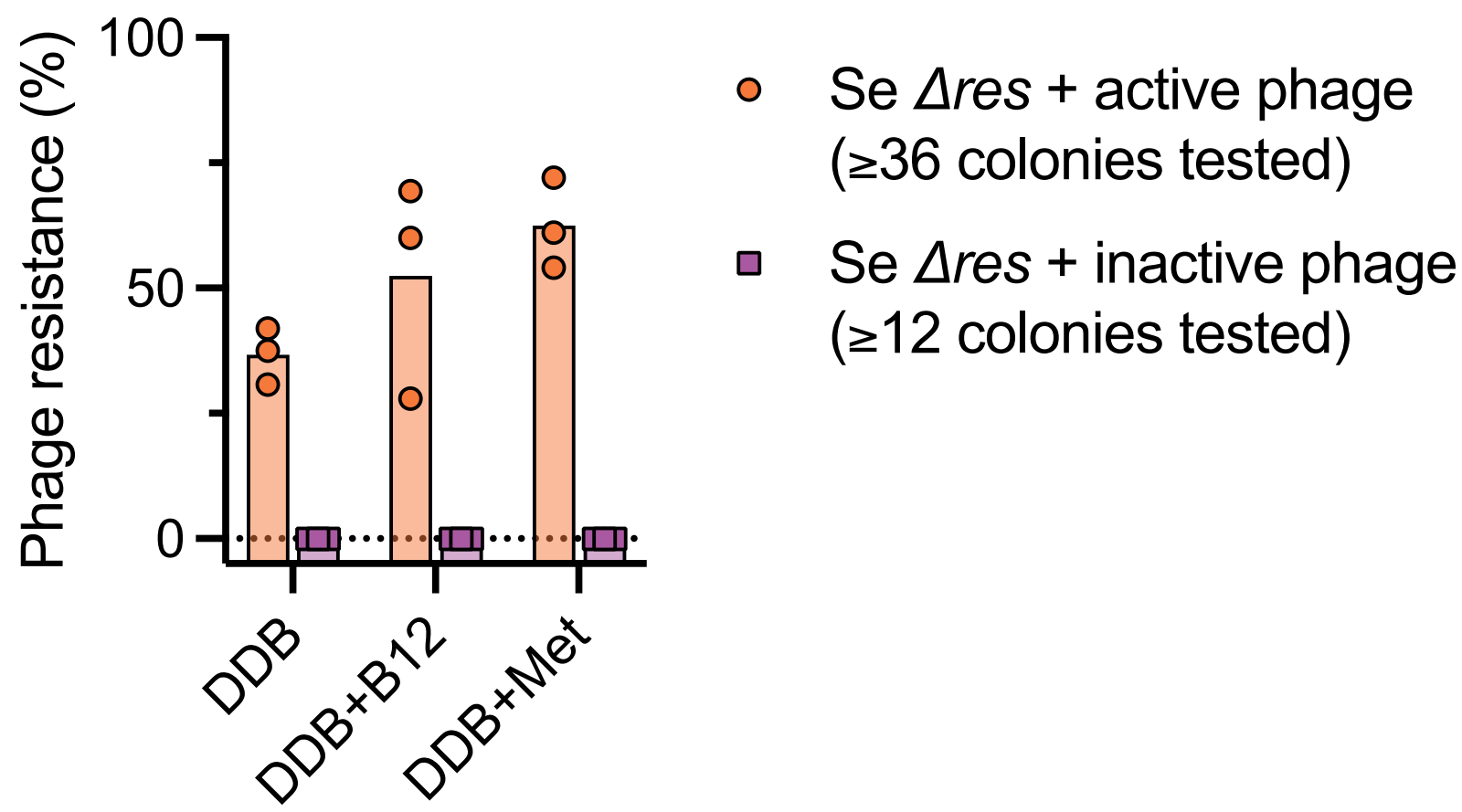

Fig. S10. Phage resistance of Se Δ*res* colonies measured after 24 h in mixed-culture experiments.

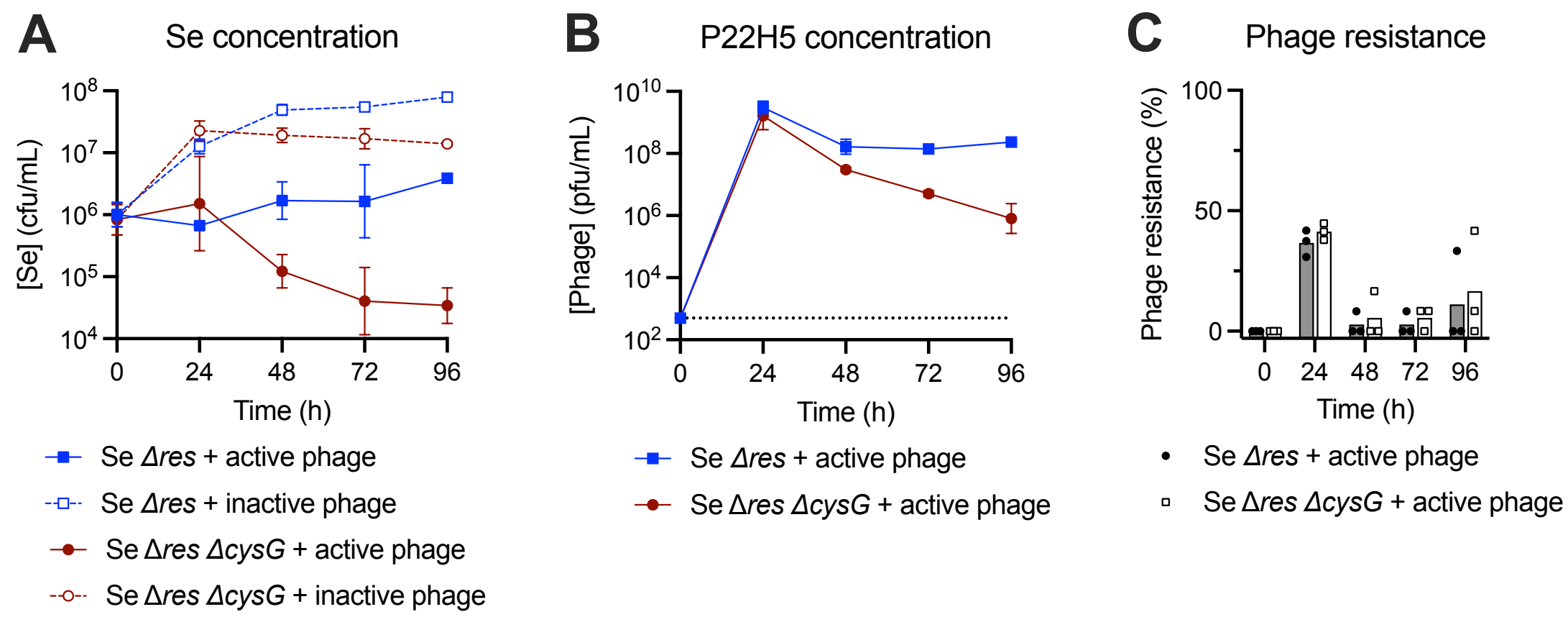

Fig. S11. Producer strains Se Δ*res* and Se Δ*res* Δ*cysG* were infected with active or heat-inactivated phage P22H5 (MOI = 10^{-4}) in mixed-culture experiments. (A) The number of cells (CFU/mL), (B) phage titers and (C) phage resistance of producer colonies were measured over time.

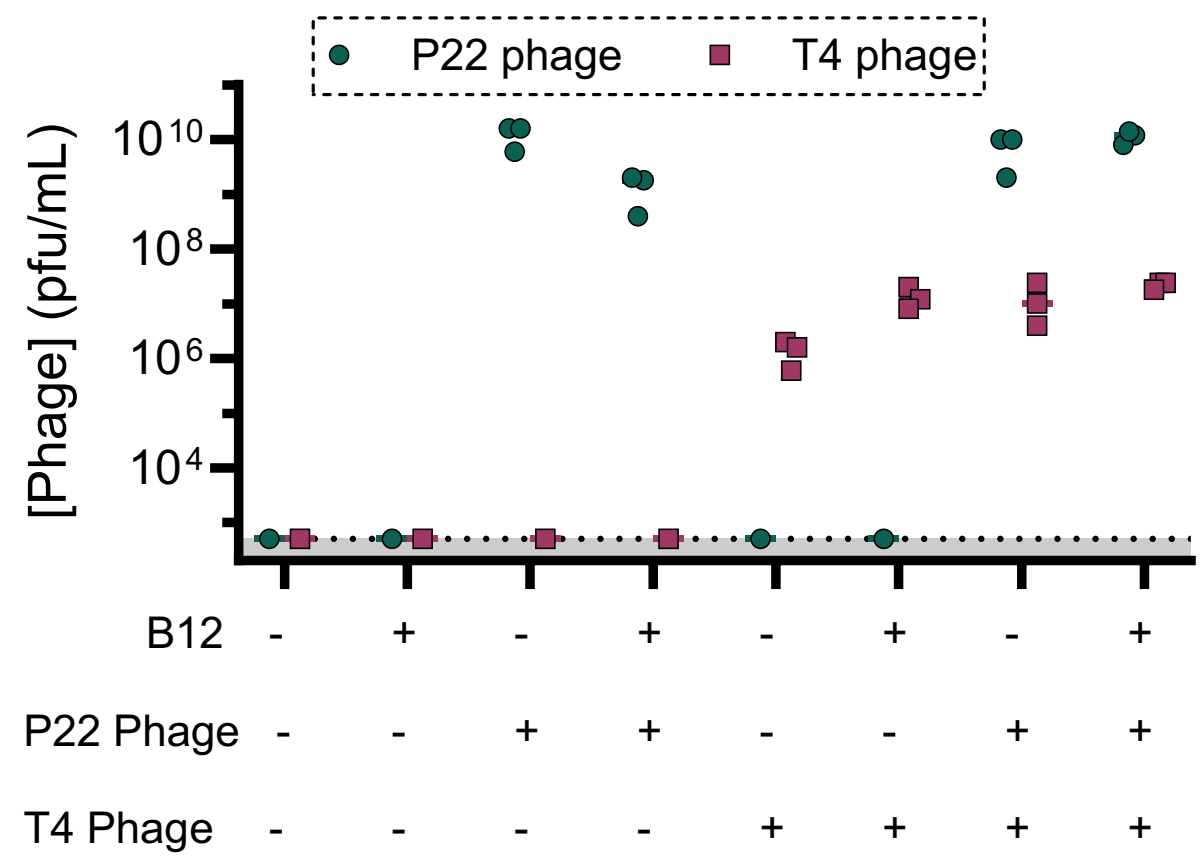

Fig. S12. P22 and T4 phages titers quantified after 24 h in mixed-culture experiments.

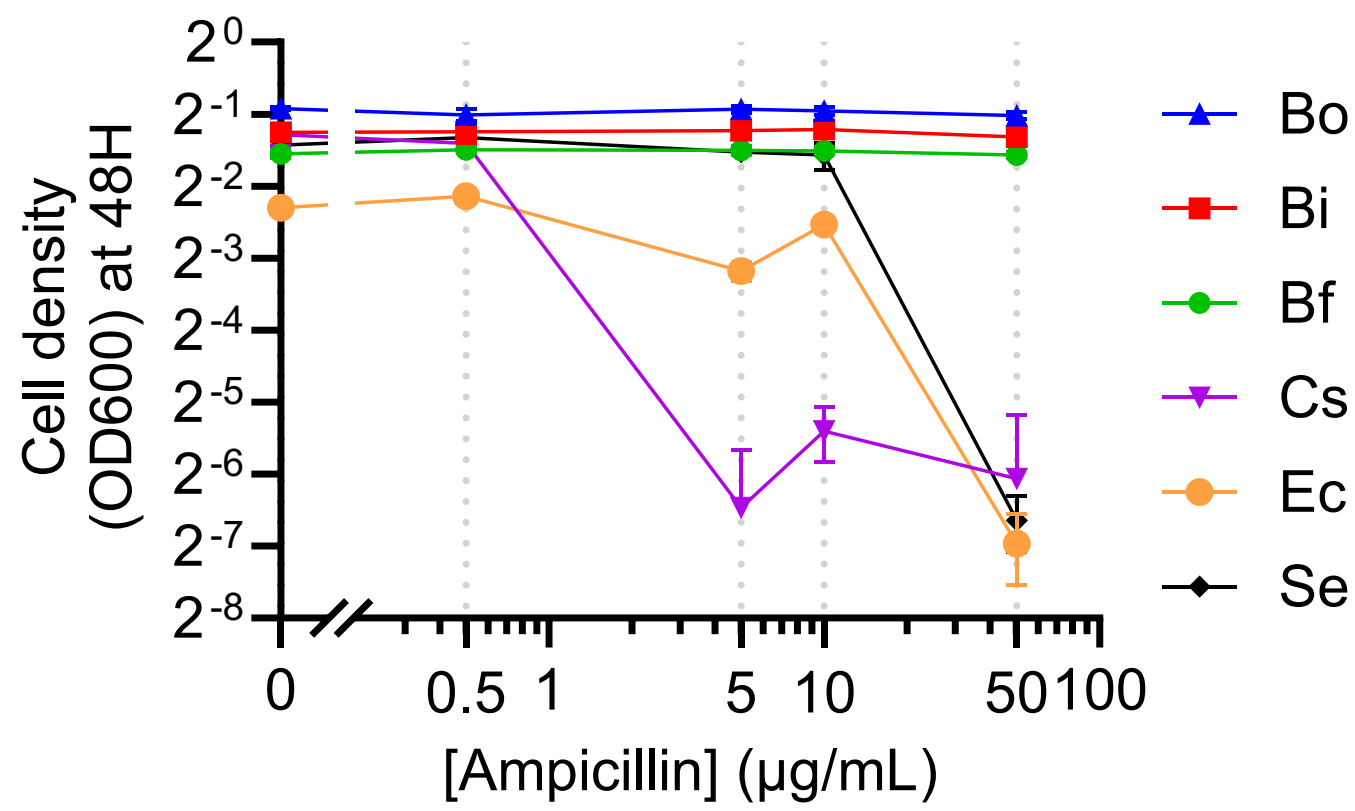

Fig. S13. **Ampicillin titration curves of a bacterial consortium in B12-supplemented DDB medium**.

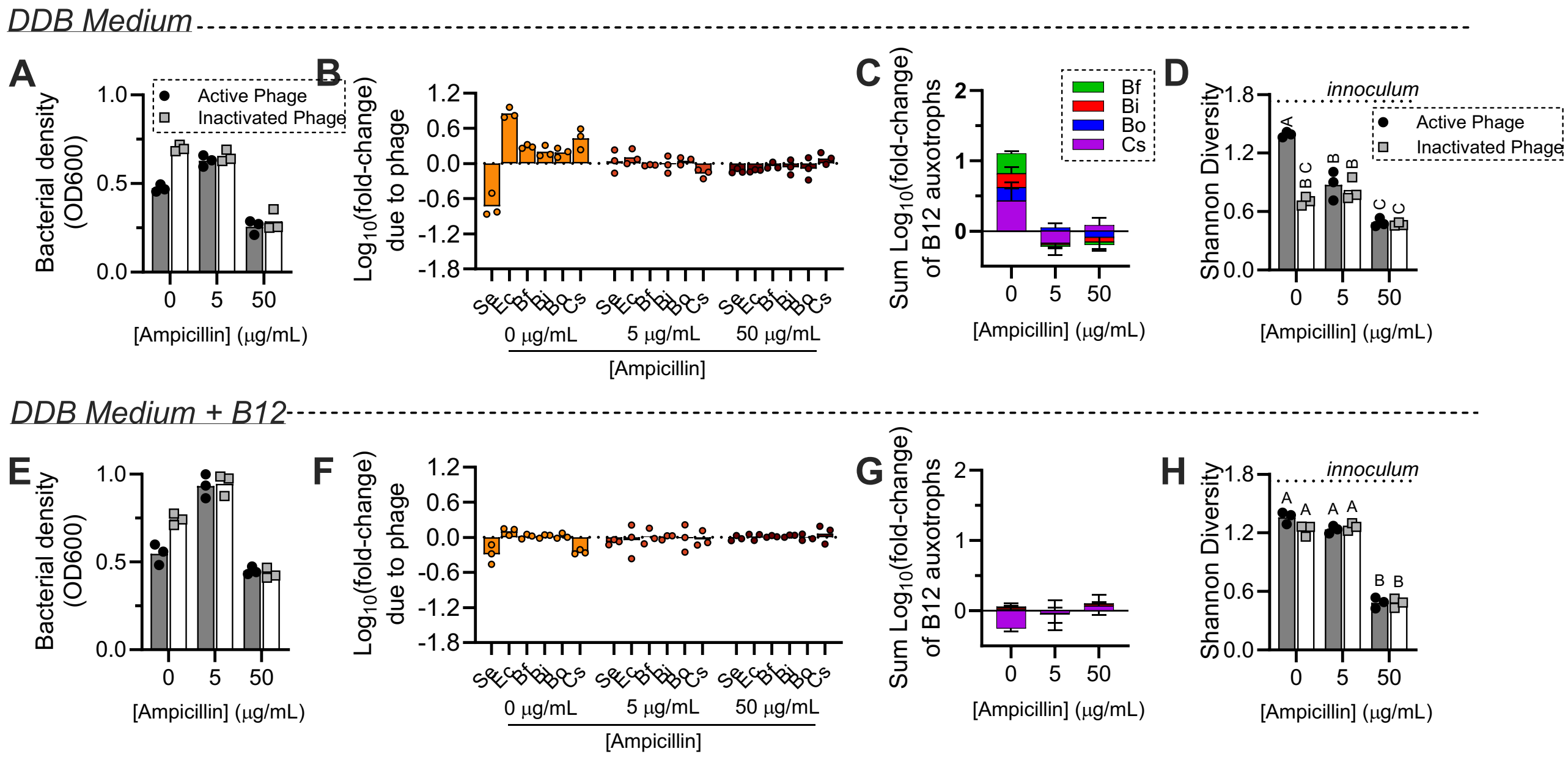

Fig S14. Phage-mediated alteration of a bacterial community composition is abrogated by ampicillin treatment. The bacterial community (Ec, Bf, Bi, Bo, Cs and Se Δ*res*) was cultured as describe in Figure 4 with low (5 μg/mL) or high (50 μg/mL) concentrations of ampicillin. Active and inactivated phages P22H5 were inoculated at an MOI of 0.0001. (**A**) The bacterial densities measured by absorbance (600 nm) at 24h. (**B**) The log fold-change in concentration of each bacterium due to active versus heat-inactivated P22H5 phage in mixed cultures (Se, Ec, Bf, Bi, Bo, Cs). (**C**) The sum of log fold-change of the Commensal-Users due to P22H5. (**D**) The Shannon Index of the community. The dashed horizontal line represents the Shannon Index of the inoculum. **(E-H)** Corresponding analysis were performed on the same culture in DDB medium supplemented with 5 ng/mL of B12. Statistical significance was determined by two-way ANOVA or a mixed-effects model with a Bonferroni multiple comparisons test. Unique letter codes indicate statistical significance with P<0.05.

SUPPLEMENTAL TABLES

Table S1. Strains, plasmids and oligonucleotides used in this study.

**Bacterium annotation**	**Genotype**	**Phenotype related to B12 and methionine**	**Resistance(s)**	**Source**
Producer strains				
Salmonella enterica Typhimurium 14028s				
Se Δ*res*	Δ*res*::Cm	Produce B12 **and** methionine	Cm^r, $P22H5^s$	Michael McClelland, University of California Irvine
Se Δ*res* Δ*cbiB*	Δ*cbiB* Δ*res*::Cm	Does not produce B12 but produce its precursors, produces methionine	Cm^r, $P22H5^s$	This work
Se Δ*res* Δ*cysG*	Δ*cysG* Δ*res*::Cm	Does not produce B12, produces methionine	Cm^r, $P22H5^s$	This work
User strains				
Salmonella enterica Typhimurium 14028s				
Se Δ*rfbP* Δ*metE* Δ*cbiB*	Δ*metE* Δ*cbiB* Δ*rfbP::kan*	Require B12 **or** methionine for growth	Kan^r, $P22H5^r$	This work
Se Δ*rfbP* Δ*metE* Δ*metH*	Δ*metE* Δ*metH* Δ*rfbP*::Kan	Require methionine for growth	Kan^r, $P22H5^r$	This work
Se Δ*rfbP*	Δ*rfbP*::Kan	Does not require B12 or methionine for growth	Kan^r, $P22H5^r$	Michael McClelland, University of California Irvine
Escherichia coli K-12 BW25113				
Ec Δ*metE*	Δ*metE*::Kan	Require B12 **or** methionine for growth	Kan^r	Keio Collection (PMID: 16738554)
Ec Δ*metE* Δ*metH*	Δ*metE*::Kan Δ*metH*	Require methionine for growth	Kan^r	This work
Ec Δ*ars*	Δ*arsB*::Kan	Does not require B12 or methionine for growth	Kan^r	Keio Collection (PMID: 16738554)
Bacteroides fragilis JCM 11017				
Bf	-	Require B12 or methionine for growth	-	JCM
Bacteroides intestinalis JCM 13265T				
Bi	-	Require B12 or methionine for growth	-	JCM
Bacteroides ovatus JCM 5824T				

Bo	-	Require B12 or methionine for growth	-	JCM
Clostridium sporogenes ATCC 17866				
Cs	-	Require B12 or methionine for growth	-	ATCC
Escherichia coli Nissle 1917				
Ec	-	Does not require B12 or methionine for growth	-	Lynn Bry, Brigham and Womens Hospital

**Phage(s)**	**Genotype**	**Relevant characteristic or description**	**Source**
Salmonella bacteriophages			
P22H5	c2-mutant	Virulent phage	Kelly Hughes, University of Utah
P22 HT105/1 int-201		Used for generalized transduction.	Michael McClelland, University of California Irvine
Escherichia coli bacteriophages			
T4		Virulent phage	ATCC 11303-B4
P1		Used for generalized transduction.	Pamela Silver, Harvard Medical School

**Plasmids**	**Relevant characteristic or description**	**Resistance(s)**	**Source or reference**
pKD46	Carries lambda red recombinase under the control of an araBAD promoter.	Amp^r	Pamela Silver, Harvard Medical School
pCP20	Carries flipase recombinase under the control of a temperature inducible promoter.	Amp^r, Cm^r	Pamela Silver, Harvard Medical School

Cm^r, chloramphenicol resistant; Kan^r, kanamycin resistant; Amp^r, ampicillin resistant; P22H5$^{s/r}$, sensitive/resistant to the phage P22H5; JMC, Japan Collection of Microorganisms; ATCC, American Type Culture Collection.

**Oligonucleotides**	**Sequence (5'_3')**
PCR primers	
Generation of Δ*metH* deletion mutant in Salmonella	
FD_metH	CAGTCCGCATCGTAACCCAGGTTCGGCGCGAGCCACCGCTCAAC-ATTCCGGGGATCCGTCGACC
RV_metH	GTGGGTGGCAAGCTGAACATGTCTCATGTTGCCCGTTGTTCTCTT-GTGTAGGCTGGAGCTGCTTC
S. Typhimurium 14028s mutant verification	
FD_metH	ATCACAGGATGCCCGATAGCG
RV_metH	ACGGCGTCAGGTTCTCCTTATC
FD_metE	CGTTGGCTTCCTTGCTTACC
RV_metE	GGATGGGTTATTTCGCCTGAC
FD_cysG	GGTGTCGTATTCATGGGATTGG

RV_cysG	CAGGTCAGGTCAAATTGAGTGC
FD_cbiB	CTGGCGAAACGGTAATCAGGACG
RV_cbiB	GCGTGTTACAGGAATGGACTGG
FD_res	CTTTCACCGAGTCAGGATTTACCG
RV_res	GCTGGCTGTCAGGCTTATCG
FD_rfbP	CGGTATAACCACGGCTTTCG
RV_rfbP	CCTGAGTTACGCTGCTATGC
E.coli K-12 BW25113 mutant verification	
FD_metH	TCATGTTGCGTGGTGGTCG
RV_metH	AGAGACAGGGCACTCATTGAGC
FD_metE	CGTTGGCTGCGTTTCTCCAC
RV_metE	GCGGGCTGCTAATGCGAAAG

FD, forward; RV, reverse
